# Graft‐versus‐leukaemia immunity is retained following treatment with post‐transplant cyclophosphamide alone or combined with tocilizumab in humanised mice

**DOI:** 10.1002/cti2.1497

**Published:** 2024-03-15

**Authors:** Chloe Sligar, Ellie Reilly, Peter Cuthbertson, Kara L Vine, Katrina M Bird, Amal Elhage, Stephen I Alexander, Ronald Sluyter, Debbie Watson

**Affiliations:** ^1^ Molecular Horizons and School of Chemistry and Molecular Bioscience University of Wollongong Wollongong NSW Australia; ^2^ Illawarra Health and Medical Research Institute Wollongong NSW Australia; ^3^ The Children's Hospital at Westmead Westmead NSW Australia

**Keywords:** graft‐versus‐leukaemia, humanised mice, post‐transplant cyclophosphamide, tocilizumab, xenogeneic graft‐versus‐host disease

## Abstract

**Objectives:**

Donor haematopoietic stem cell transplantation treats leukaemia by inducing graft‐versus‐leukaemia (GVL) immunity. However, this benefit is often mitigated by graft‐versus‐host disease (GVHD), which is reduced by post‐transplant cyclophosphamide (PTCy) alone or combined with tocilizumab (TOC) in humanised mice. This study established a preclinical humanised mouse model of GVL and investigated whether PTCy alone or combined with TOC impacts GVL immunity.

**Methods:**

NOD‐*scid*‐IL2Rγ^null^ mice were injected with 2 × 10^7^ human peripheral blood mononuclear cells (hPBMCs) on day 0 and with 1 × 10^6^ THP‐1 acute myeloid leukaemia cells on day 14. In subsequent experiments, mice were also injected with PTCy (33 mg kg^−1^) or Dulbecco's phosphate buffered saline (PBS) on days 3 and 4, alone or combined with TOC or control antibody (25 mg kg^−1^) twice weekly for 28 days. Clinical signs of disease were monitored until day 42.

**Results:**

Mice with hPBMCs from three different donors and THP‐1 cells showed similar survival, clinical score and weight loss. hCD33^+^ leukaemia cells were minimal in mice reconstituted with hPBMCs from two donors but present in mice with hPBMCs from a third donor, suggesting donor‐specific GVL responses. hPBMC‐injected mice treated with PTCy alone or combined with TOC (PTCy + TOC) demonstrated prolonged survival compared to control mice. PTCy alone and PTCy + TOC‐treated mice with hPBMCs showed minimal hepatic hCD33^+^ leukaemia cells, indicating sustained GVL immunity. Further, the combination of PTCy + TOC reduced histological damage in the lung and liver.

**Conclusion:**

Collectively, this research demonstrates that PTCy alone or combined with TOC impairs GVHD without compromising GVL immunity.

## Graphical abstract

Donor haematopoietic stem cell transplantation treats leukaemia by inducing graft‐versus‐leukaemia (GVL) immunity. However, this benefit is often mitigated by graft‐versus‐host disease (GVHD), which is reduced by post‐transplant cyclophosphamide (PTCy) alone or combined with tocilizumab (TOC) in humanised mice. This study established a preclinical humanised mouse model of GVL and demonstrates that PTCy alone or combined with TOC impairs GVHD without compromising GVL immunity.

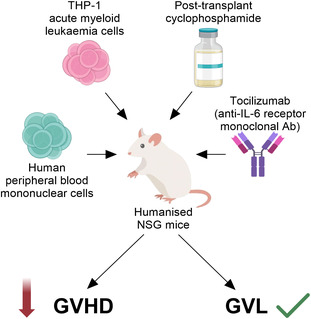

## Introduction

Donor haematopoietic stem cell transplantation (HSCT) is a curative therapy for haematological malignancies, including leukaemia, which are resistant to conventional chemotherapy and radiotherapy.^1^ Donor HSCT aims to generate the graft‐versus‐leukaemia (GVL) effect, where the alloreactive donor T cells eliminate residual host malignant cells.^2^ However, a common and often fatal side effect of this treatment is graft‐versus‐host disease (GVHD), which in the absence of post‐transplant treatments, occurs in 40–60% of donor HSCT recipients.^3^ GVHD occurs when alloreactive donor T cells recognise the host's healthy tissues as foreign and mount a potent inflammatory immune response against them. GVHD results in severe damage, primarily to the skin, liver, gastrointestinal tract^4^ and lungs,^5^ and is fatal in 15–30% of cases.^3^

The GVL effect was first observed over 60 years ago,^6^ yet despite years of preclinical and clinical studies, the precise mechanism required to achieve GVL immunity without promoting GVHD has not been uncovered. It is thought that the GVL effect is mediated predominantly by T cells but can also be mediated by natural killer (NK) cells and B cells, and cytokines produced by these cells.^2^, ^7^, ^8^ The pathogenesis of GVHD is also mediated predominantly by T cells. As such, one therapeutic strategy for GVHD prevention is the use of post‐transplant cyclophosphamide (PTCy), a cytotoxic compound often administered at 50 mg kg^−1^ on days 3 and 4 post‐donor HSCT.^9^ PTCy has been shown to deplete or reduce the proliferation of T cells in both allogeneic and humanised mouse models.^10^, ^11^, ^12^

While clinical use of PTCy reduces the incidence of GVHD to 30% following donor HSCT,^13^ the precise mechanism by which it ameliorates GVHD and impacts reactive donor T cells remains poorly understood. Studies on the effect of PTCy on the GVL response are limited and the potential detrimental impact of PTCy on disease relapse is of concern. Initial clinical studies observed relatively high rates of relapse with administration of PTCy.^14^, ^15^ However, a recent study in humanised mice found that PTCy reduced GVHD by limiting xenoreactive T cell proliferation and favoring the recovery of donor regulatory T cells (Tregs), while GVL effects were reduced but not abrogated.^16^ Nevertheless, it remains unclear whether the GVL effect is maintained when PTCy is given as a GVHD therapeutic.

The pleiotropic cytokine interleukin (IL)‐6 has a primary role in T cell differentiation^17^ and GVHD development.^18^ In the presence of IL‐6 and transforming growth factor beta, naive T cells develop into T helper (Th) 17 cells, which can exacerbate GVHD.^19^ However, in the absence of IL‐6, the same naive T cells develop into Tregs, which are protective against GVHD.^20^ Tocilizumab (TOC) is a humanised monoclonal antibody directed against the IL‐6 receptor that inhibits both classical and trans‐signalling.^21^ Clinically, the use of TOC as a prophylactic therapy for GVHD has had limited success, where a single 8 mg kg^−1^ dose on day –1 with cyclosporine/methotrexate did not significantly reduce acute GVHD or improve survival outcomes.^22^ However, we have previously shown that combining PTCy with TOC delays GVHD onset, reduces weight loss and improves survival in humanised mice^23^ but the impact of this combination therapy on GVL immunity is yet to be explored.

The current study aimed to establish a humanised mouse model of GVL and determine the impact of PTCy alone and in combination with TOC on GVL responses, human immune cells and cytokines using this preclinical model.

## Results

### Survival, clinical score and weight loss are similar between NSG mice injected with hPBMCs from different donors and THP‐1 leukaemia cells

**Figure 1:**
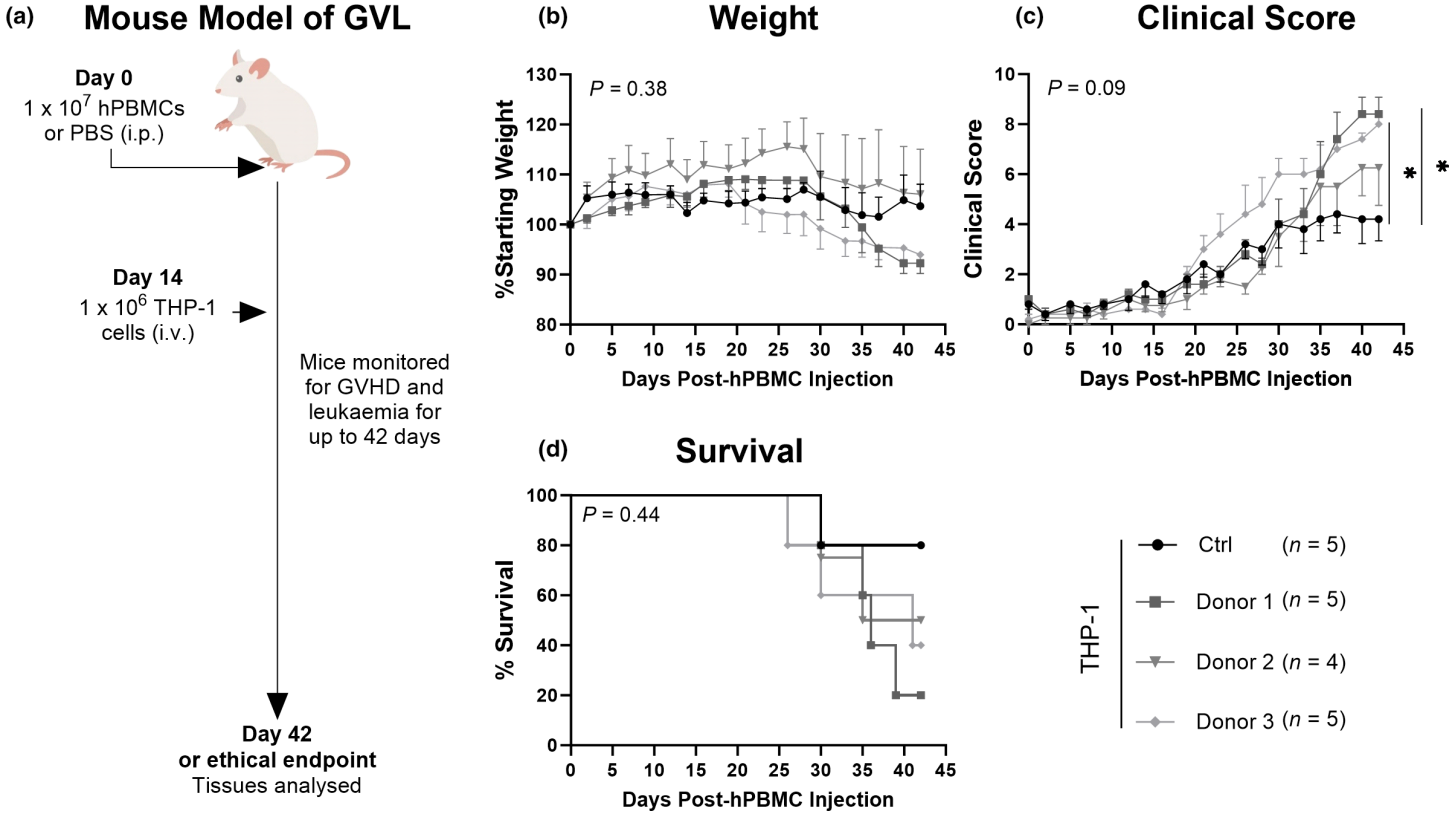
Survival, clinical score and weight loss are similar between NSG mice injected with hPBMCs from different donors and THP‐1 leukaemia cells. **(a)** Schematic overview of humanised graft‐versus leukaemia (GVL) mouse model. NOD‐*scid*‐IL2Rγ^null^ (NSG) mice were injected intraperitoneally (i.p.) with 1 × 10^7^ human peripheral blood mononuclear cells (hPBMCs) from donors 1 (D1), 2 (D2) and 3 (D3) or PBS on day 0 and injected intravenously (i.v.) with 1 × 10^6^ THP‐1 cells on day 14. Mice were monitored at least thrice weekly for clinical signs of graft‐versus‐host disease (GVHD) and leukaemia including **(b)** weight, **(c)** clinical score and **(d)** survival over 42 days. Data are represented as the mean ± SEM. Data are from one experiment. Significance was analysed using **(b, c)** two‐way ANOVA and **(d)** log‐rank (Mantel‐Cox) tests. *P* < 0.05 (*).

Our initial objective was to establish a model of GVL and assess the efficacy of hPBMCs obtained from different donors in generating a GVL response to THP‐1 leukaemia cells in humanised mice. NSG mice were injected with 1 × 10^7^ hPBMCs (from three healthy donors) or Dulbecco's phosphate buffered saline (PBS) (THP‐1 only control mice). On day 14, mice were injected with 1 × 10^6^ THP‐1 leukaemia cells. This resulted in four groups: donor 1 (D1), donor 2 (D2), donor 3 (D3) and control (Ctrl) mice, which were monitored at least thrice weekly for up to 42 days for GVHD and leukaemia development (Figure 1a) using a standardised scoring system (Table 1).

**Table 1:** Clinical scoring criteria for the assessment of GVHD and leukaemia in humanised mice

Score^a^	0	1	2	3^b^
Activity	Cage exploration prior to removal of lid. Activity is normal, standing on hind limbs and climbing shelters	Remains ≥ 2 min in nest/shelter when lid is removed. Some reduction in activity but stands on hind limbs and actively explores cages and shelters	Slow to move/explore cages and shelters. Commences exploring when shelters are removed without being nudged or handled. Extends hind limbs partly when walking, that is, not in a sitting/shuffling position when moving	Remains stationary when shelters are removed or after shuffling to another spot. Does not explore cage until nudged or handled. Walking is slow with pronounced shortened gait/shuffle with minimal/no hind limb extension
Posture	Normal	Hunching noted at rest only	Hunching noted at rest and movement, with a hunched back when walking	Severe hunching/very hunched when stationary. Nose is close to the ground when resting. Prominent hunch/kyphosis of spine when moving
Hind limb function	Normal gait and can stand on hind limbs	Not seen standing on hind limbs or ascending shelters	Not using hind limb(s) normally or mild paresis	Hind limb paresis/paralysis with hind limb(s) dragging
Fur	Normal coat, smooth and glossy	Mild to moderate ruffling but no skin visible	Severe ruffling/hair loss (balding) < 50% of mouse surface area	≥ 50% of mouse surface area with hair loss (balding)
Skin integrity	Normal	Scaling of paws/tail	Scaling at additional areas, that is, around eyes, nose and muzzle	Areas of denuded skin with loss of surface layers and possible wounds/ulcers/craters
Chronic weight loss^c^	< 5%	5–9.9%	10–14.9%	≥ 15%
Acute weight loss^d^	< 5%	5–6.9%	7–14.9%	≥ 15% or ≥ 13% for ≥ 48 h

^a^ NSG mice were injected with 1 or 2 × 10^7^ hPBMCs and 1 × 10^6^ THP‐1 cells and monitored for up to 42 days post‐hPBMC injection for GVHD and leukaemia development by scoring for activity, posture, hind limb function, fur, skin integrity and chronic or acute weight loss.

^b^ Mice were euthanised if a score of 3 in any category was reached.

^c^ Chronic weight loss was determined as percentage loss from starting weight (weight on day 0).

^d^ Acute weight loss was determined as > 15% weight loss over any 7‐day period.

Weight loss was similar between all groups up to day 20, at which point, D3 mice began to lose weight (Figure 1b). D2 and D1 mice began to lose weight from day 28, whereas Ctrl mice maintained a steady weight until endpoint. However, there was no significant difference between groups (*P* = 0.38). At endpoint, the weight of D1 and D3 mice was lower than that of D2 mice; however, this was not significant (*P* = 0.52 and *P* = 0.61, respectively).

D1 and D3 mice had low (≤ 3) clinical scores until days 28 and 16, respectively, which then steadily increased until endpoint (Figure 1c). D2 and Ctrl mice also had low clinical scores up until day 26, which increased until endpoint but to a lesser extent than the former two groups. At endpoint, the difference between all groups approached significance (*P* = 0.09), with the clinical score of D1 and D3 mice significantly higher than that of Ctrl mice (*P* = 0.02 and *P* = 0.03, respectively). The clinical score of D2 mice was similar to that of D1 and D3 mice (*P =* 0.60 and *P* = 0.70, respectively). Survival was similar between all four groups (*P* = 0.44), with a median survival time (MST) > 42 days for control mice, 36 days for D1, 38.5 days for D2 and 41 days for D3 (Figure 1d).

### Donor‐specific GVL responses were observed in the livers of humanised NSG mice

**Figure 2:**
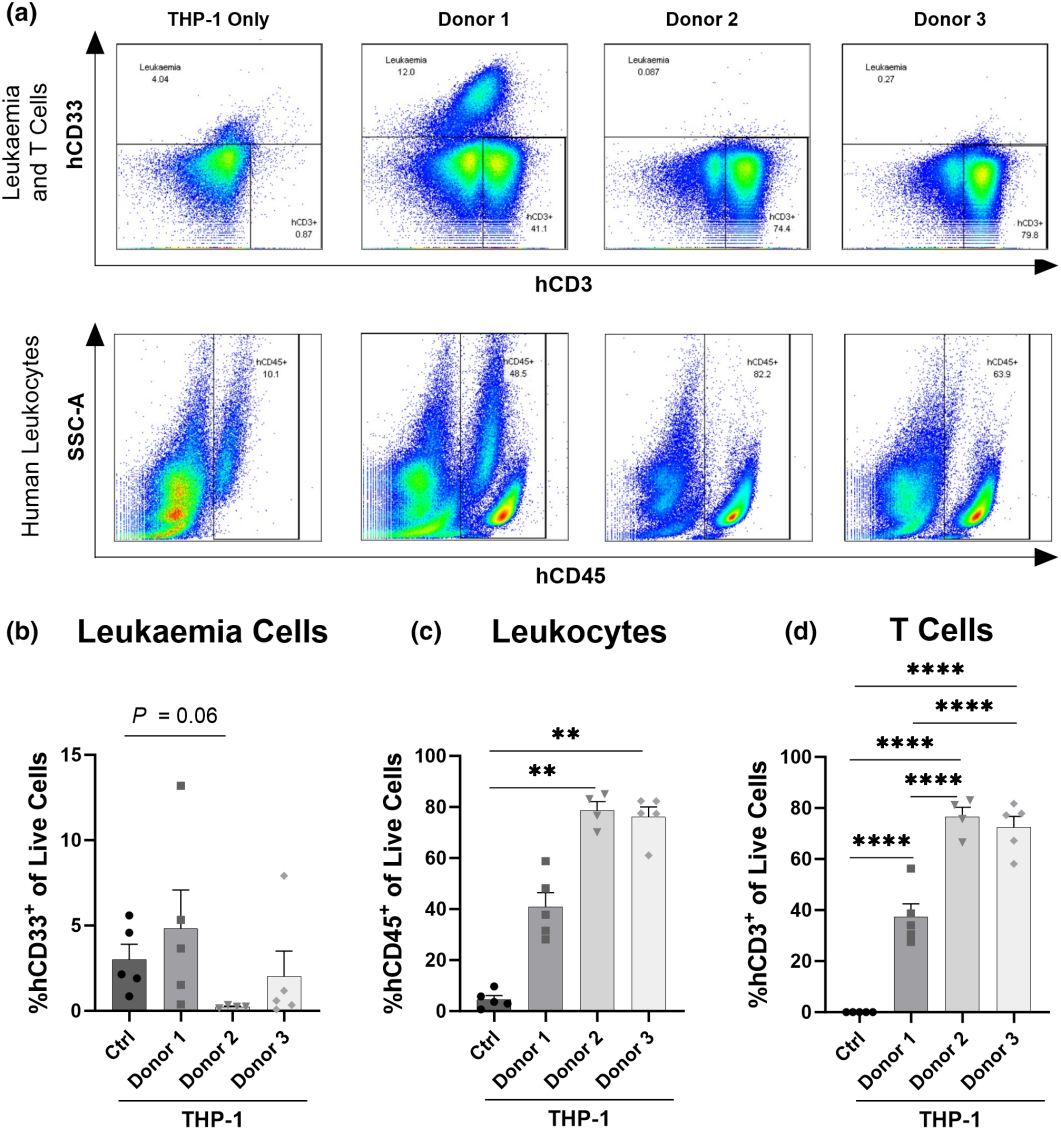
The GVL response to THP‐1 leukaemia cells is donor specific in the livers of humanised NSG mice. Livers from NOD‐*scid*‐IL2Rγ^null^ (NSG) mice injected intraperitoneally (i.p.) with 1 × 10^7^ human peripheral blood mononuclear cells (hPBMCs) from donors 1 (D1), 2 (D2) and 3 (D3) or PBS and intravenously (i.v.) with 1 × 10^6^ THP‐1 cells on day 14 were collected at day 42 or ethical endpoint. Human (h) leukaemia and leukocyte subsets were analysed by flow cytometry using **(a)** a consistent gating strategy. Live cells were gated based on Zombie NIR staining (not shown), leukaemia cells and T cells were gated as hCD33 and hCD3, respectively, and leukocytes were gated using side‐scatter area (SSC‐A) and hCD45. Proportions of **(b)** hCD33^+^hCD3^−^ leukaemia cells, **(c)** hCD45^+^ leukocytes and **(d)** hCD3^+^hCD33^−^ T cells were gated and analysed as a proportion of live cells. **(b, c)** Data are presented as the mean ± SEM. Data are from one experiment. Symbols represent individual mice. Significance was determined using either **(d)** one‐way ANOVA or **(b, c)** Kruskal–Wallis tests. *P* < 0.0001 (****), *P* < 0.01 (**).

THP‐1 cells primarily engraft in the liver of non‐irradiated NSG mice (Supplementary figure 1). Additionally, the liver is one of the primary target organs of GVHD and previous studies have shown that human leukocytes engraft in the livers of humanised NSG mice.^12^, ^24^ Therefore, engraftment of THP‐1 cells and human leukocytes in NSG mice injected with hPBMCs and THP‐1 cells or THP‐1 cells only was assessed by flow cytometry in the liver (Figure 2a–d) and spleen (Supplementary figure 2). THP‐1 cells are a monocytic cell line that express hCD33.^25^ Although hPBMCs contain monocytes that also express hCD33, these cells do not engraft in NSG mice.^26^ Therefore, this marker was used to identify engraftment of leukaemia cells (hCD3^−^hCD33^+^) (Figure 2a).

The proportions of leukaemia cells were similar (3.0 ± 0.89% vs 4.8 ± 2.26%) between Ctrl and D1 mice (*P* > 0.99) (Figure 2b). Conversely, the proportions of leukaemia cells were reduced by 92% in D2 mice (0.2 ± 0.04 vs 3.0 ± 0.89), which approached statistical significance (*P* = 0.06) and by 33% in D3 mice (2.0 ± 1.5%) (*P* > 0.99) compared to Ctrl mice.

The proportions of human leukocytes (consisting of both THP‐1 cells and hPBMCs) were low in Ctrl mice (4.6 ± 1.5%) (Figure 2c). Compared to Ctrl mice, the proportions of hCD45^+^ were nine‐fold higher in D1 mice, though this was not significant (*P* = 0.96), and 17‐fold higher in D2 and D3 mice (*P* = 0.006 and *P* = 0.007, respectively). D1 mice exhibited proportions of hCD45^+^ cells 47% lower than D2 and D3 mice; however, this difference was not significant (*P* = 0.28 and *P* = 0.38, respectively).

As expected, hCD3^+^ T cells were not detected in Ctrl mice (Figure 2d). Notably, the proportions of hCD3^+^ T cells in D1 mice (37.4 ± 5.1%) were approximately 50% lower than D2 and D3 mice (76.6 ± 3.7% and 72.4 ± 4.3%, respectively) (*P* < 0.0001 for both groups), while proportions between D2 and D3 mice were similar (*P* = 0.87). Taken together, this data indicates that the GVL response is donor‐specific in this model, with D1 hPBMCs unable to elicit a GVL response.

### NSG mice injected with hPBMCs from donors able to elicit a GVL response have a lower hCD4^+^:hCD8^+^ ratio, higher hTh17 cells and hTh17:hTreg ratio

**Figure 3:**
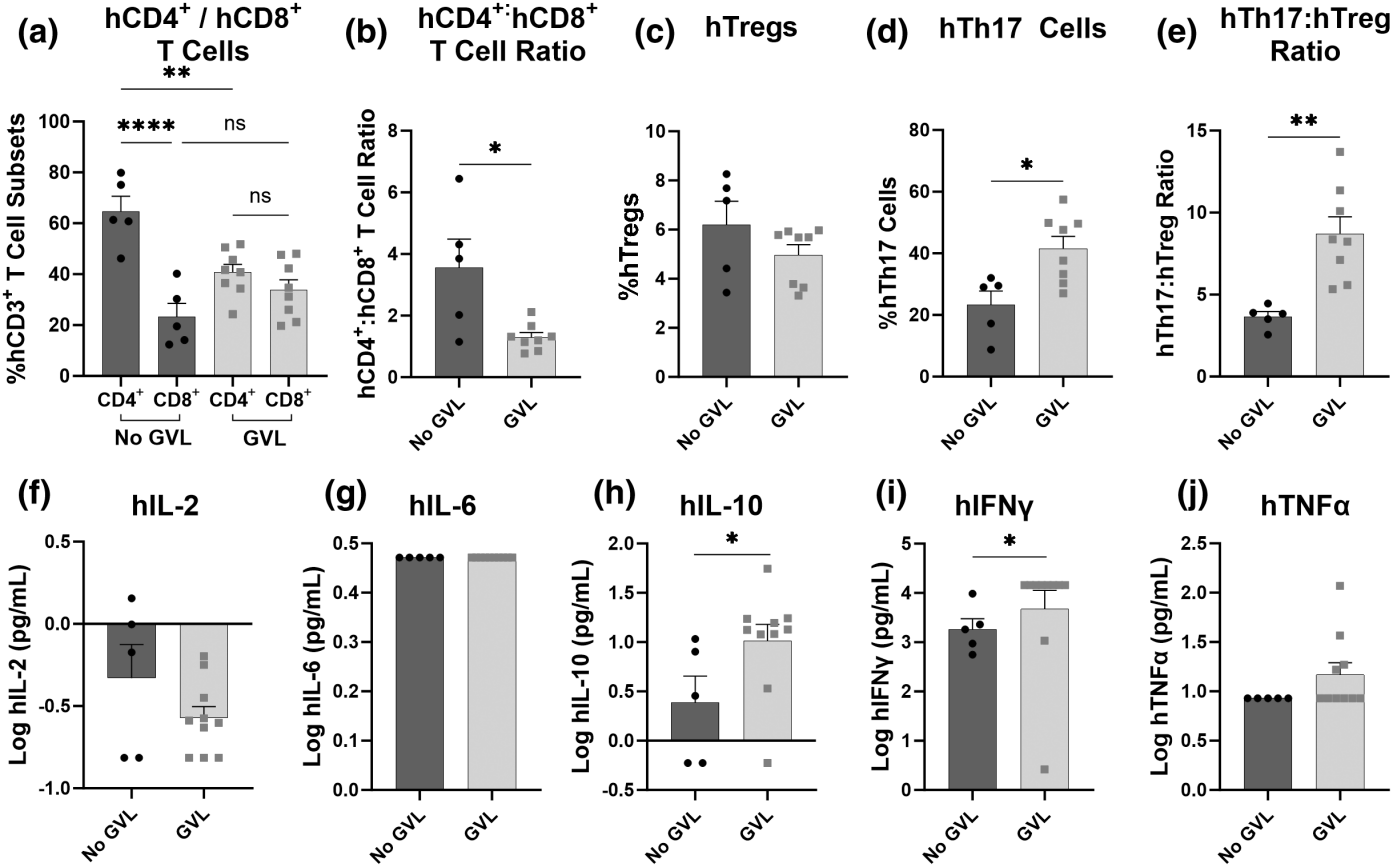
Donors that elicit GVL responses to THP‐1 leukaemia cells in humanised mice have reduced liver CD4^+^:CD8^+^ T cell ratios and increased Th17:Treg ratios. Livers from NOD‐*scid*‐IL2Rγ^null^ (NSG) mice injected intraperitoneally (i.p.) with 1 × 10^7^ human peripheral blood mononuclear cells (hPBMCs) from donors 1 (D1), 2 (D2) and 3 (D3) and intravenously (i.v.) with 1 × 10^6^ THP‐1 cells were collected at day 42 or ethical endpoint and analysed by flow cytometry using an established gating strategy.^23^ Data from mice injected with hPBMCs from the donor that was unable to elicit a GVL response (No GVL) were compared to those injected with hPBMCs from donors that did establish a GVL response (GVL). The proportion of hCD45^+^, mCD45^+^ and hCD3^+^ populations were identified before analysing **(a)** hCD4^+^ and hCD8^+^ T cells, **(b)** hCD4^+^:hCD8^+^ T cell ratio, **(c)** hCD4^+^hCD25^+^hCD127^low^ regulatory T cells (hTregs), **(d)** hCD4^+^hCD161^+^hCD39^+^ T helper 17 (hTh17) cells and **(e)** hTh17:hTreg ratio. At day 42 or ethical endpoint, sera were isolated from the blood of NSG mice. The concentration of **(f)** human (h) interleukin (IL)‐2, **(g)** IL‐6, **(h)** IL‐10, **(i)** interferon‐gamma (IFNγ) and **(j)** tumor necrosis factor‐alpha (TNFα) was assessed using a LEGENDplex assay. Data are presented as the mean ± SEM and are from one experiment. Symbols represent individual mice. Significance was determined using either **(a)** one‐way ANOVA, **(b, d–g)** unpaired Student's *t*‐tests‐ or **(c, h–j)** Mann–Whitney *U* tests. *P* < 0.0001 (****), *P* < 0.01 (**), *P* < 0.05 (*), not significant (ns).

GVHD and the GVL response are both primarily mediated by donor alloreactive T cells.^2^, ^27^ To determine whether differences in T cell subsets contributed to the lack of GVL response for D1, immune cell subsets in the liver (Figure 3a–e) and spleen (Supplementary figure 2) were assessed by flow cytometry. Data from mice injected with donor (D1) hPBMCs that were unable to elicit a GVL response (No GVL mice) were compared to those injected with hPBMCs from donors (D2, D3) that did induce a GVL response (GVL mice). The proportions of hCD4^+^ T cells were 2.8‐fold greater than the proportions of hCD8^+^ T cells in No GVL mice (*P* < 0.0001), whereas proportions of hCD4^+^ and hCD8^+^ T cells were similar in GVL mice (*P* = 0.61) (Figure 3a). As such, the hCD4^+^:hCD8^+^ T cell ratio was 2.7‐fold greater in No GVL compared to GVL mice (*P* = 0.01) (Figure 3b).

Tregs reduce the severity of GVHD through the suppression of pathogenic T cells^28^ without having a significant impact on the GVL response.^29^ The proportions of hTregs (hCD4^+^hCD25^+^hCD127^low^) were similar between No GVL and GVL mice (*P* = 0.28) (Figure 3c). In contrast to Tregs, Th17 cells exacerbate GVHD.^19^ Compared to No GVL mice, the proportions of hTh17 (hCD4^+^hCD39^+^hCD161^+^) cells were 1.7‐fold higher in GVL mice (*P* = 0.01) (Figure 3d), corresponding to a hTh17:hTreg ratio that was 2.3‐fold greater in GVL mice (*P* = 0.003) (Figure 3e). These variations in immune cell subsets were similar in the spleens of these mice; however, there was a 63.5% decrease in splenic hTregs in GVL mice (*P* = 0.0004) (Supplementary figure 2).

To further investigate potential mechanisms behind the GVL response in this model, serum hIL‐2, hIL‐6, hIL‐10, human interferon‐gamma (hIFNγ) and human tumour necrosis factor‐alpha (hTNFα) concentrations were examined (Figure 3f–j). The concentration of hIL‐2 was similar between No GVL and GVL mice (*P* = 0.18) (Figure 3f), as was the concentration of hIL‐6 (*P* > 0.99) (Figure 3g). Notably, compared to No GVL mice, the concentration of hIL‐10 was 2.6‐fold higher (*P* = 0.02) (Figure 3h) and the concentration of hIFNγ was 1.2‐fold higher in GVL mice (*P* = 0.03) (Figure 3i). The concentration of TNFα was similar between groups (*P* = 0.23) (Figure 3j).

### PTCy reduces weight loss and prolongs time to GVHD onset in NSG mice injected with hPBMCs and THP‐1 leukaemia cells

After establishing a humanised mouse model of GVL, we next aimed to examine whether the GVL response was maintained following treatment with PTCy at days 3 and 4. PTCy has been shown to reduce GVHD in a humanised mouse model^12^, ^23^, ^30^ but studies examining the impact of PTCy on GVL immunity and the mechanisms behind GVL responses are limited.

**Figure 4:**
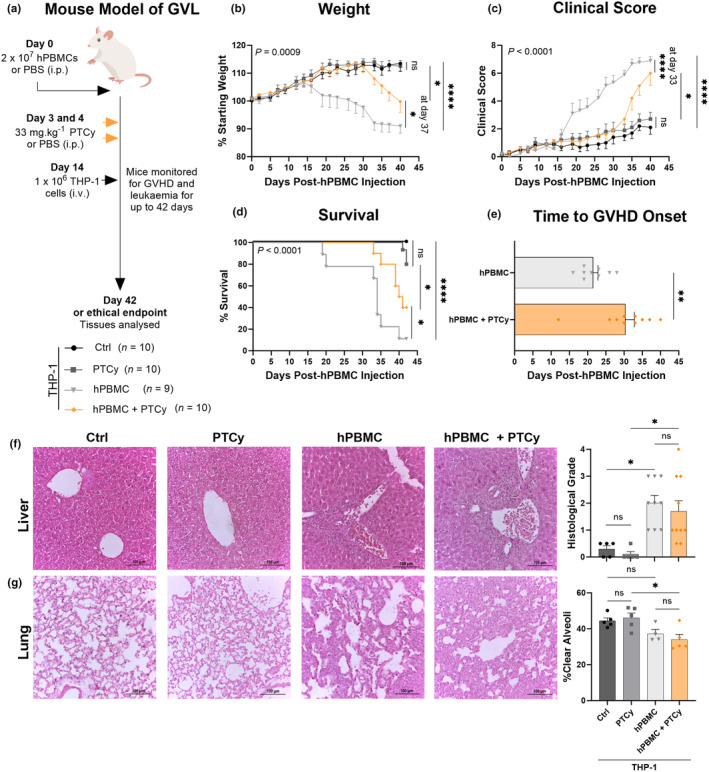
PTCy reduces weight loss and improves survival and clinical score in NSG mice injected with hPBMCs and THP‐1 leukaemia cells. **(a)** Schematic overview of humanised graft‐versus‐leukaemia (GVL) mouse model. NOD‐*scid*‐IL2Rγ^null^ (NSG) mice were injected intraperitoneally (i.p.) with 2 × 10^7^ human peripheral blood mononuclear cells (hPBMCs) (*n* = 2 donors; D2 and D3) or PBS on day 0. On days 3 and 4, mice were injected i.p. with cyclophosphamide (33 mg kg^−1^) (PTCy) or PBS and on day 14, mice were injected intravenously (i.v.) with 1 × 10^6^ THP‐1 cells. Mice were monitored at least thrice weekly for clinical signs of GVHD and leukaemia including **(b)** weight, **(c)** clinical score and **(d)** survival over 42 days. **(e)** Time to GVHD onset (defined as a clinical score ≥ 3) was also determined. **(f, g)** Tissues from mice were sectioned and stained with haematoxylin and eosin. Histological damage was assessed for **(f)** liver using a standardised grading system and **(g)** lung as the percentage of clear alveoli area of total lung area. Images are representative of up to 10 mice per treatment group. Data are represented as the mean ± SEM. Data are from two independent experiments. Significance was analysed using **(b, c)** two‐way ANOVA, **(d)** the log‐rank (Mantel‐Cox) test, **(e)** the unpaired Student's *t*‐test, **(f)** the Kruskal–Wallis test or **(g)** one‐way ANOVA. *P* < 0.0001 (****), *P* < 0.01 (**), *P* < 0.05 (*), not significant (ns).

On day 0, NSG mice were injected with 2 × 10^7^ hPBMCs (from D2 and D3), double that of the above experiments to account for PTCy‐induced depletion of hPBMCs, or PBS. On days 3 and 4 post‐hPBMC injection, mice were injected with either cyclophosphamide (33 mg kg^−1^) (PTCy) or PBS and on day 14, all mice were injected with 1 × 10^6^ THP‐1 cells (Figure 4a). This resulted in four treatment groups: Control (Ctrl; THP‐1 only), PTCy (THP‐1 + PTCy), hPBMC (THP‐1 + hPBMCs) and hPBMC + PTCy (THP‐1 + hPBMCs + PTCy). Mice were monitored at least thrice weekly for up to 42 days using a standardised scoring system (as above).

Ctrl and PTCy mice continued to gain weight until endpoint with no significant difference between the two groups (*P* = 0.99) (Figure 4b). Conversely, hPBMC mice gained weight until day 14 before continuously losing weight until endpoint and hPBMC + PTCy mice continued gaining weight until day 31 when mice progressively lost weight until endpoint. At day 37, the weight loss of hPBMC + PTCy mice was significantly improved compared to hPBMC mice (*P* = 0.04) and was significantly worse in hPBMC and hPBMC + PTCy mice compared to Ctrl mice (*P* < 0.0001 for both groups).

The clinical score of Ctrl and PTCy mice remained similarly low (≤ 3) until endpoint (*P* = 0.82) (Figure 4c). The clinical score of hPBMC mice began to increase from day 18 and continued to increase until endpoint. In hPBMC + PTCy mice, the rise in clinical score was delayed until day 33 and was significantly reduced compared to hPBMC mice at this time point (*P* < 0.0001); however, the clinical score of hPBMC + PTCy mice continued to rise until endpoint where the difference between these two groups was no longer significant (*P* = 0.77). Compared to Ctrl mice, the clinical scores of hPBMC and hPBMC + PTCy mice were significantly higher at endpoint (*P* < 0.0001 and *P* = 0.007, respectively).

Ctrl and PTCy mice had similar survival (MST > 42 days), with the majority of mice surviving until endpoint (*P* = 0.12) (Figure 4d). Compared to hPBMC mice (MST = 34 days), survival outcomes were significantly prolonged in hPBMC + PTCy mice (MST = 40.5 days) (*P* = 0.03). Survival outcomes of both hPBMC and hPBMC + PTCy mice were significantly shorter than Ctrl mice (*P* < 0.001 and *P* = 0.003, respectively). Given the clinical data above, time to GVHD onset, defined as a clinical score ≥ 3,^23^ was evaluated. Time to GVHD onset was significantly delayed in hPBMC + PTCy mice compared to hPBMC mice (30 days vs 21 days, respectively) (*P* = 0.006) (Figure 4e).

The liver and lung are two target organs of GVHD in humanised NSG mice^24^ and in a previous study, qualitative analysis revealed that PTCy reduced histological GVHD in the liver.^12^ In the current study, histological damage was examined and quantified in the liver (Figure 4f) and lung (Figure 4g). Histopathology in the liver was similarly low (histological grade: 0–0.5) in Ctrl and PTCy mice (*P* > 0.99). Conversely, moderate to high histological evidence of disease was observed in the livers of hPBMC and hPBMC + PTCy mice, which was similar between the two groups (*P* > 0.99). Compared to Ctrl and PTCy mice, histological liver pathology was significantly higher in hPBMC and hPBMC + PTCy mice (*P* = 0.02 and *P* = 0.01, respectively). Together, these results indicate that the small scores observed in Ctrl mice are likely because of THP‐1 cells being identified as infiltrates and the histological damage observed is because of hPBMC‐induced GVHD or GVL effects, which is not impacted by PTCy in this model.

The mean percentage of clear alveoli space (an inverse measure of lung GVHD) in the lung of Ctrl and PTCy mice was similar (44.4–46.1%) (*P* = 0.96). In hPBMC and hPBMC + PTCy mice, the mean percentage of clear alveoli space was reduced by 16% and 26%, compared to control and PTCy mice, respectively (*P* = 0.2 and *P* = 0.01, respectively). This indicates that the reduction in open alveoli space is not impacted by PTCy in this model.

### PTCy does not impact the GVL response and reduces the hCD4^+^:hCD8^+^ T cell ratio in NSG mice injected with hPBMCs and THP‐1 leukaemia cells

**Figure 5:**
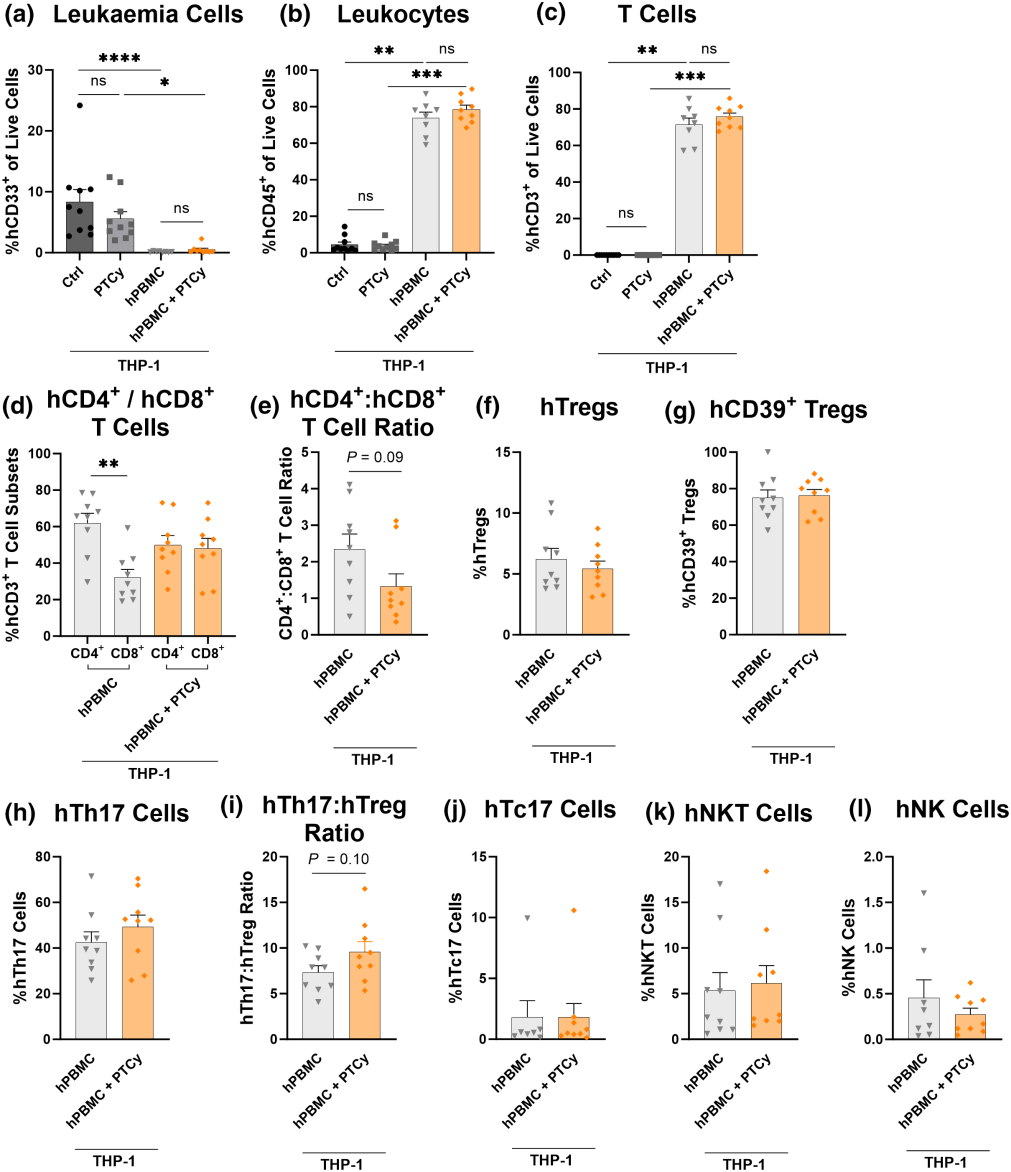
PTCy does not impact the GVL response to THP‐1 leukaemia cells in the liver of humanised mice and reduces the hCD4^+^:hCD8^+^ T cell ratio. Livers from NOD‐*scid*‐IL2Rγ^null^ (NSG) mice injected i.p. with 2 × 10^7^ human peripheral blood mononuclear cells (hPBMCs) (*n* = 2 donors; D2 and D3) or PBS on day 0, i.p. with cyclophosphamide (33 mg kg^−1^) (PTCy) or PBS on days 3 and 4, intravenously (i.v.) with 1 × 10^6^ THP‐1 cells on day 14 were examined by flow cytometry using the gating strategy shown in Figure 2a to identify human (h) leukaemia cells and leukocyte subsets at endpoint. **(a)** hCD33^+^ leukaemia cells, **(b)** hCD45^+^ leukocytes and **(c)** hCD3^+^ T cells. Proportions of subsequent subsets were examined using an established gating strategy.^23^ The proportion of hCD45^+^, mCD45^+^ and hCD3^+^ populations were identified before gating **(d)** hCD4^+^ and hCD8^+^ T cell subsets, **(e)** hCD4^+^:hCD8^+^ T cell ratio, **(f)** hCD4^+^hCD25^+^hCD127^low^ regulatory T cells (hTregs), **(g)** hCD39^+^ hTregs, **(h)** hCD4^+^hCD161^+^hCD39^+^ T helper 17 (hTh17) cells, **(i)** hTh17:hTreg ratio, **(j)** hCD8^+^hCD161^high^ (hTc17) cells, **(k)** hCD3^+^hCD56^+^ natural killer T (hNKT) cells and **(l)** hCD3^−^hCD56^+^ natural killer (hNK) cells. Data are presented as the mean ± SEM. Symbols represent individual mice. Data are from two independent experiments. Significance was tested using **(a–c)** Kruskal–Wallis tests, **(d)** one‐way ANOVA, **(g–j)** unpaired Student's *t*‐tests or **(e, f, k, l)** Mann–Whitney *U*‐tests. *P* < 0.001 (***), *P* < 0.01 (**), *P* < 0.05 (*), not significant (ns).

In humanised mice, PTCy can alter the proportion of human leukocytes which may play a role in GVL immunity.^12^ To determine whether hPBMCs were still able to reduce leukaemia cells when PTCy is given as a treatment for GVHD, engraftment of THP‐1 cells and human leukocytes in Ctrl, PTCy, hPBMC and hPBMC + PTCy mice in the liver (Figure 5) and spleen (Supplementary figure 3) was assessed by flow cytometry using the above gating strategy (Figure 2a).

In the liver, the proportions of leukaemia cells were similar between Ctrl and PTCy mice (8.3 ± 2.0% vs 5.6 ± 1.2%) (*P* > 0.99) (Figure 5a), indicating PTCy administered prior to THP‐1 injection does not impact THP‐1 cells directly. Leukaemia cells were minimal and similar in hPBMC and hPBMC + PTCy mice (0.2 ± 0.1% vs 0.5 ± 0.2%) (*P* > 0.99). Compared to Ctrl mice, the proportions of leukaemia cells were significantly reduced by 97% in hPBMC mice (*P* < 0.0001). Together, these results demonstrate that PTCy does not impact the GVL response in these mice.

As expected, Ctrl and PTCy mice had similarly low proportions of hCD45^+^ cells (4.4 ± 1.4% vs 4.0 ± 0.8%) (*P* > 0.99) (Figure 5b), which represent leukaemia cells. hPBMC and hPBMC + PTCy mice had similarly high proportions of hCD45^+^ cells (65.7 ± 8.6% vs 78.5 ± 2.4%) (*P* > 0.99). Further, hCD3^+^ T cells were not detected in Ctrl or PTCy mice, given that these mice were not injected with hPBMCs. Conversely, the proportions of hCD3^+^ T cells were similarly high in hPBMC and hPBMC + PTCy mice (71.5 ± 3.5% vs 75.8 ± 2.1%) (*P* > 0.99) (Figure 5c). These results indicate that PTCy does not impact the total proportion of hCD45^+^ leukocytes, which are predominantly T cells, in this model.

We have previously observed an increased hCD4^+^:hCD8^+^ T cell ratio following PTCy treatment in the spleens of humanised mice.^12^ However, in a subsequent study, this variation in hCD4 and hCD8 proportions in both spleens and livers was not observed.^23^ Therefore, in the current study, the effect of PTCy on human T cell subsets in this model was assessed by flow cytometry of livers from hPBMC and hPBMC + PTCy mice using an established gating strategy.^23^ In hPBMC mice, the proportions of hCD4^+^ T cells were significantly higher than the proportions of hCD8^+^ T cells (*P* = 0.0015) (Figure 5d). Conversely, proportions of hCD4^+^ and hCD8^+^ T cells were similar in hPBMC + PTCy mice (*P* = 0.99). As such, compared to hPBMC mice, the hCD4^+^:hCD8^+^ T cell ratio was reduced by 46% in hPBMC + PTCy mice (*P* = 0.09) (Figure 5e). This reduction was also observed in the spleens of hPBMC + PTCy mice (Supplementary figure 3). Given that increased hCD4^+^:hCD8^+^ T cell ratios are associated with clinical GVHD in humanised mice,^31^ these results provide a possible mechanism by which PTCy reduces GVHD.

Tregs are reportedly resistant to depletion by PTCy due to their expression of aldehyde dehydrogenase^32^; however, a decrease in Tregs following PTCy in the spleens of humanised mice has previously been observed.^12^ Therefore, proportions of hTregs were examined. In the liver, the proportions of hTregs were similar between hPBMC and hPBMC + PTCy mice (6.2 vs 5.4%) (*P* = 0.48) (Figure 5f) as were the proportions of hCD39^+^ hTregs (75.1 vs 76.3%) (*P* = 0.83), which are highly suppressive and can influence GVHD^33^ (Figure 5g).

In the liver, proportions of hTh17 cells were 1.2‐fold higher in hPBMC + PTCy mice (42.5 vs 49.2%) (*P* = 0.35) (Figure 5h) and the hTh17:hTreg ratio was 1.3‐fold higher in hPBMC + PTCy mice (7.4 vs 9.6) (*P* = 0.10) (Figure 5i) compared to hPBMC mice. While this increase did not reach statistical significance in the livers of mice, it did reach statistical significance in the spleen (Supplementary figure 3), indicating that PTCy may promote hTh17 differentiation, or Treg/Th17 plasticity in this model. GVHD can be induced by cytotoxic T (Tc) 17 (hCD8^+^hCD161^high^) cells.^34^ However, the proportions of hTc17 cells were similar between both groups (1.8 vs 1.8%) (*P* = 0.76) (Figure 5j).

Natural killer T (NKT) (hCD3^+^hCD56^+^) cells can suppress GVHD^35^ but do not appear to interfere with GVL immunity.^36^, ^37^ Proportions of hNKT cells were similar between hPBMC and hPBMC + PTCy mice (5.3 vs 6.1%) (*P* = 0.43) (Figure 5k). Moreover, NK cells can suppress GVHD in mice^38^ while promoting GVL responses.^39^ However, proportions of hNK (hCD3^−^hCD56^+^) cells were similar between these groups (0.5 vs 0.3%) (*P* = 0.98) (Figure 5l). Finally, serum hIL‐2, hIL‐6, hIL‐10, hIFNγ and hTNFα concentrations were examined in these mice and were found to be similar between both groups (Supplementary figure 4). These results indicate that PTCy does not impact hNKT cells, hNK cells or cytokines which may be important in generating GVL responses.

### TOC combined with PTCy prolongs time to GVHD onset in NSG mice injected with hPBMCs and THP‐1 leukaemia cells

**Figure 6:**
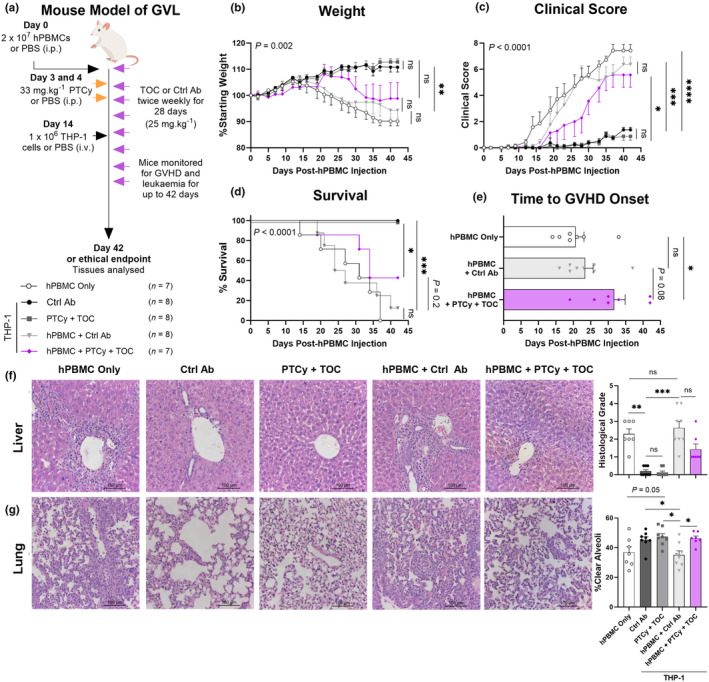
PTCy combined with TOC improves survival, delays GVHD onset and reduces histological GVHD in the liver and lung of NSG mice injected with hPBMCs and THP‐1 leukaemia cells. **(a)** Schematic overview of humanised graft‐versus‐leukaemia (GVL) mouse model. NOD‐*scid*‐IL2Rγ^null^ (NSG) mice were injected intraperitoneally (i.p.) with 2 × 10^7^ human peripheral blood mononuclear cells (hPBMCs) (*n* = 2 donors; D2 and D3) or PBS on day 0 and tocilizumab (TOC) or control antibody (Ctrl Ab) (25 mg kg^−1^) twice weekly for 28 days. On days 3 and 4, mice were injected i.p. with cyclophosphamide (33 mg kg^−1^) (PTCy) or PBS and on day 14, mice were injected intravenously (i.v.) with 1 × 10^6^ THP‐1 cells or PBS (hPBMC only). Mice were monitored at least thrice weekly for clinical signs of graft‐versus‐host disease (GVHD) and leukaemia including **(b)** weight, **(c)** clinical score and **(d)** survival over 42 days. **(e)** Time to GVHD onset (defined as a clinical score ≥ 3) was also determined. **(f, g)** Tissues from mice were sectioned and stained with haematoxylin and eosin. Histological damage was assessed for **(f)** liver using a standardised grading system and **(g)** lung as the percentage of clear alveoli area of total lung area. Images are representative of up to 8 mice per treatment group. Data are presented as the mean ± SEM. Data are from two independent experiments. Significance was analysed using **(b, c)** two‐way ANOVA, **(d)** the log‐rank (Mantel‐Cox) test, **(e, g)** one‐way ANOVA or **(f)** the Kruskal–Wallis test. *P* < 0.0001 (****), *P* < 0.001 (***), *P* < 0.01 (**), *P* < 0.05 (*), not significant (ns).

In clinical trials, a single dose of TOC on day −1 prior to HSCT (with cyclosporine/methotrexate prophylaxis) did not significantly reduce acute GVHD.^22^ However, we have previously shown that combination therapy with TOC and PTCy delays GVHD onset, reduces weight loss and improves survival in a humanised mouse model of GVHD.^23^ As such, we examined whether the GVL response was maintained when this therapy was given as GVHD prevention. NSG mice were injected with 2 × 10^7^ hPBMCs (from D2 and D3) or PBS and injected i.p. with either TOC (25 mg kg^−1^) or a control antibody (Ctrl Ab) (25 mg kg^−1^) twice weekly for 28 days. On days 3 and 4 post‐hPBMC injection, mice were injected with either cyclophosphamide (33 mg kg^−1^) (PTCy) or PBS. On day 14, all mice (excluding hPBMC only mice) were injected i.v. with 1 × 10^6^ THP‐1 cells (Figure 6a). This resulted in five treatment groups: hPBMC only (hPBMC only: hPBMCs + Ctrl Ab), control Ab (Ctrl Ab: THP‐1 + Ctrl Ab), PTCy + TOC (THP‐1 + PTCy + TOC), hPBMC + control Ab (THP‐1 + hPBMCs + Ctrl Ab) and hPBMC + PTCy + TOC (THP‐1 + hPBMCs + PTCy + TOC). Mice were monitored at least thrice weekly for up to 42 days using a standardised scoring system (as above).

hPBMC only mice lost weight continuously from day 15 until endpoint (Figure 6b). Conversely, Ctrl Ab and PTCy + TOC mice gained weight until endpoint with no significant difference between groups (*P* = 0.89). Similar to hPBMC only mice, hPBMC + Ctrl Ab mice lost weight continuously from day 15 until endpoint, whereas hPBMC + PTCy + TOC mice began losing weight from day 25; however, there was no difference between these groups (*P* = 0.96).

The clinical score of hPBMC only mice increased sharply until endpoint from day 12 (Figure 6c). However, the clinical scores of Ctrl Ab and PTCy + TOC mice remained similarly low until endpoint (*P* = 0.62). Similar to hPBMC only mice, the clinical scores of hPBMC + Ctrl Ab and hPBMC + PTCy + TOC mice increased sharply from days 16 and 19, respectively, until endpoint with no difference between these groups (*P* = 0.94).

All hPBMC only mice were euthanised by day 37 (MST = 31 days), whereas Ctrl Ab and PTCy + TOC mice all survived until endpoint (MST > 42 days) (Figure 6d). Survival was similar between hPBMC only and hPBMC + Ctrl Ab mice (MST = 26 days) (*P* = 0.67) and while survival was comparatively increased in hPBMC + PTCy + TOC mice (MST = 34 days), this did not reach significance (*P* = 0.20). Time to GVHD onset was similar between hPBMC only and hPBMC + Ctrl Ab mice (*P* = 0.75) (Figure 6e). Time to GVHD onset was delayed in hPBMC + PTCy + TOC significantly compared to hPBMC only (*P* = 0.02) and non‐significantly compared to hPBMC + Ctrl Ab mice (*P* = 0.08).

In Ctrl Ab and PTCy + TOC mice, histopathology was negligible and similar between both groups (*P* < 0.99), indicating that PTCy + TOC had no impact on the liver. Conversely, evidence of histological disease was present in hPBMC only mice (histological grades 1–3) and in hPBMC + Ctrl Ab (histological grades 1–4) and hPBMC + PTCy + TOC (histological grades 1–3) mice (Figure 6f). Compared to hPBMC + Ctrl Ab mice, the histological grade of hPBMC + PTCy + TOC mice was reduced by 46%; however, this was not significant (*P* > 0.99).

In the lung, hPBMC only mice showed a mean percentage of 36.9% open alveolar space, whereas Ctrl Ab and PTCy + TOC mice had similar percentages of 45.3% and 47.4%, respectively (*P* = 0.97) (Figure 6g). The mean percentage of open alveolar space of hPBMC + Ctrl Ab mice was 35.1%, similar to that of hPBMC only mice (*P* = 0.98). Comparatively, in hPBMC + PTCy + TOC mice, the mean percentage of alveoli space was significantly increased 1.3‐fold (*P* = 0.03), indicating that treatment with PTCy + TOC reduces lung pathology.

### Combination therapy with PTCy and TOC does not impact the GVL effect or immune cell subsets in NSG mice injected with hPBMCs and THP‐1 leukaemia cells

**Figure 7:**
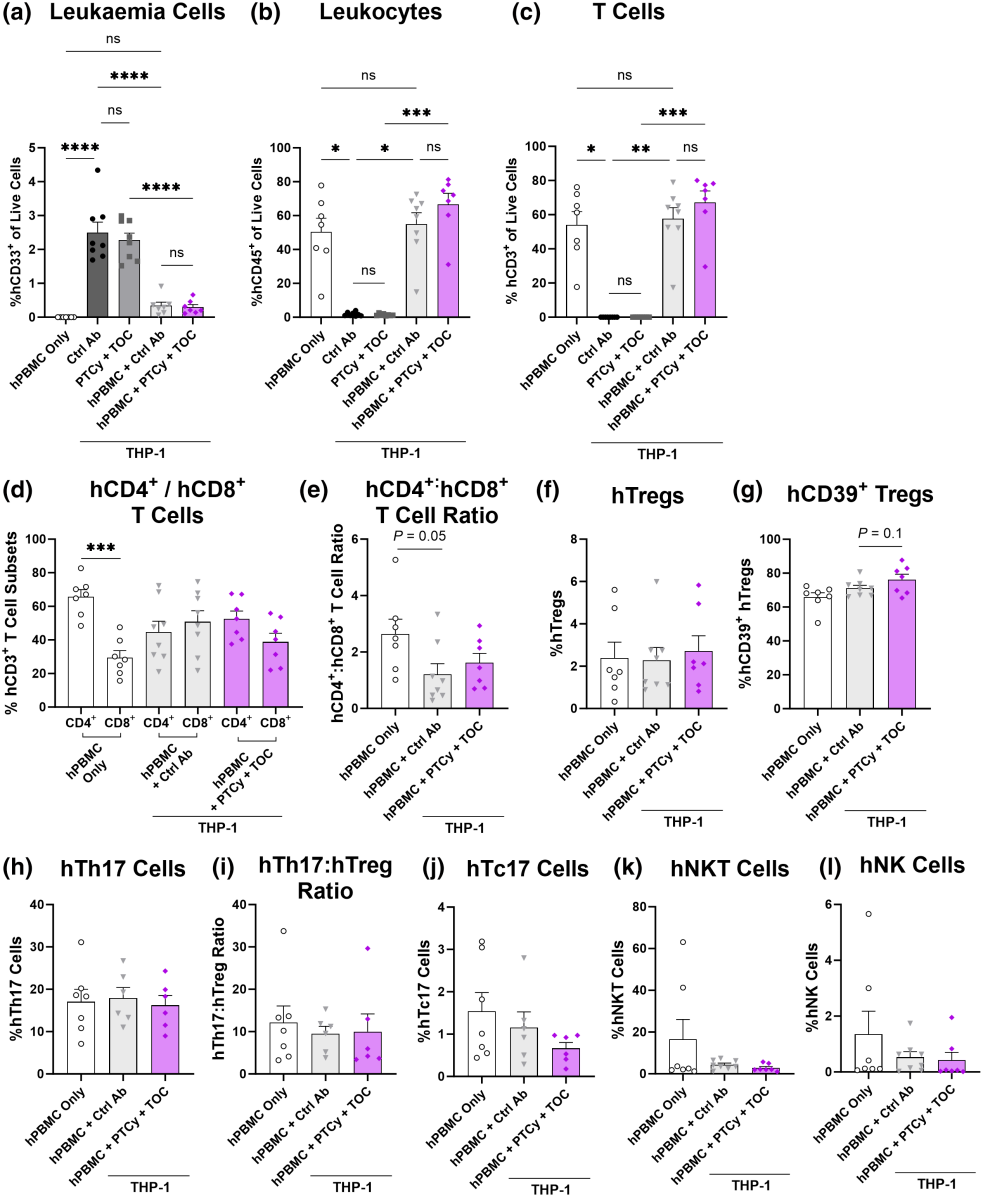
Combination therapy with PTCy and TOC does not impact the GVL response to THP‐1 leukaemia cells in the liver of NSG mice. Livers from NOD‐*scid*‐IL2Rγ^null^ (NSG) mice injected intraperitoneally (i.p.) with 2 × 10^7^ human peripheral blood mononuclear cells (hPBMCs) (*n* = 2 donors; D2 and D3) or PBS on day 0, i.p. with tocilizumab (TOC) or control antibody (Ctrl Ab) (25 mg kg^−1^) twice weekly for 28 days, i.p. with cyclophosphamide (33 mg kg^−1^) (PTCy) or PBS on days 3 and 4 and intravenously (i.v.) with 1 × 10^6^ THP‐1 cells on day 14 were examined by flow cytometry using the gating strategy shown in Figure 2a to identify human (h) leukaemia cells and leukocyte subsets at endpoint. **(a)** hCD33^+^ leukaemia cells, **(b)** hCD45^+^ leukocytes and **(c)** hCD3^+^ T cells. Proportions of subsequent subsets were examined using an established gating strategy.^23^ The proportion of hCD45^+^, mCD45^+^ and hCD3^+^ populations were identified before gating **(d)** hCD4^+^ and hCD8^+^ T cells, **(e)** hCD4^+^:hCD8^+^ T cell ratio, **(f)** hCD4^+^hCD25^+^hCD127^low^ regulatory T cells (hTregs), **(g)** hCD39^+^ hTregs, **(h)** hCD4^+^hCXCR3^−^hCD161^+^ T helper 17 (hTh17) cells, **(i)** hTh17:hTreg ratio, **(j)** hCD8^+^hCD161^high^ (hTc17) cells, **(k)** hCD3^+^hCD56^+^ natural killer T (hNKT) cells and **(l)** hCD3^−^hCD56^+^ natural killer (hNK) cells. Data are presented as the mean ± SEM. Symbols represent individual mice. Data are from two independent experiments. Significance was tested using **(d, g–l)** one‐way ANOVA or **(a–c, e, f)** Kruskal–Wallis tests. *P* < 0.0001 (****), *P* < 0.001 (***), *P* < 0.01 (**), *P* < 0.05 (*), not significant (ns).

We have previously shown that combination therapy with PTCy + TOC does not impact the engraftment of human leukocytes in humanised mice,^23^ but studies that examine the impact of TOC on GVL responses are limited. To determine whether hPBMCs were still able to reduce leukaemia cells when PTCy combined with TOC is given as a treatment for GVHD, engraftment of THP‐1 leukaemia cells and human leukocytes in the liver (Figure 7) and spleen (Supplementary figure 5) of hPBMC only, Ctrl Ab, PTCy + TOC, hPBMC + Ctrl Ab and hPBMC + PTCy + TOC mice was assessed by flow cytometry using the above gating strategy (Figure 2a).

As expected, hCD33^+^ leukaemia cells were not detected in hPBMC only mice (Figure 7a). Proportions of hCD33^+^ leukaemia cells were similar between Ctrl Ab and PTCy + TOC mice (2.5 ± 0.3% vs 2.3 ± 0.2%) (*P* = 0.91). Leukaemia cells were minimal and similar in hPBMC + Ctrl Ab and hPBMC + PTCy + TOC mice (0.3 ± 0.1% vs 0.3 ± 0.07%) (*P* = 0.99). Compared to Ctrl Ab mice, the proportions of hCD33^+^ leukaemia cells were significantly reduced by 88% in hPBMC + Ctrl Ab mice (*P* < 0.0001). This indicates that the combination treatment PTCy + TOC does not impact the GVL response.

hPBMC only mice had moderate proportions of hCD45^+^ cells (50.3 ± 8.2%) (Figure 7b). However, as expected, Ctrl Ab and PTCy + TOC mice had low and similar proportions of hCD45^+^ cells (1.8 ± 0.4% vs 1.4 ± 0.2%) (*P* > 0.99). hPBMC + Ctrl Ab and hPBMC + PTCy + TOC mice had similar proportions of hCD45^+^ cells (55.0 ± 6.7% vs 66.6 ± 6.4%) (*P* > 0.99). Proportions of hCD3^+^ T cells were high in hPBMC only mice (54.0 ± 7.8%) (Figure 7c). Given that hPBMCs were not injected, hCD3^+^ T cells were not detected in Ctrl Ab and PTCy + TOC mice. Conversely, the proportions of hCD3^+^ T cells were similarly high in hPBMC + Ctrl Ab and hPBMC + PTCy + TOC mice (57.6 ± 6.5% vs 67.2 ± 6.7%) (*P* > 0.99). This indicates that PTCy + TOC does not impact the total proportion of hCD45^+^ leukocytes or hCD3^+^ T cells in this model.

We have previously shown that PTCy + TOC increases proportions of hTregs early (day 28) in GVHD development and reduces hNK cells at endpoint.^23^ Therefore, the effect of PTCy + TOC on human leukocyte subsets was assessed by flow cytometry of livers (Figure 7) and spleens (Supplementary figure 5) from hPBMC only, hPBMC + Ctrl Ab and hPBMC + PTCy + TOC mice using an established gating strategy.^23^

In the liver of hPBMC only mice, proportions of hCD4^+^ T cells were significantly higher than those of hCD8^+^ T cells (*P* = 0.0007) (Figure 7d), which was also observed in the spleen (Supplementary figure 5). However, in hPBMC + Ctrl Ab and hPBMC + PTCy + TOC mice, proportions of hCD4^+^ and hCD8^+^ T cells were similar (*P* = 0.96 and *P* = 0.52, respectively). As such, compared to hPBMC only mice, the hCD4^+^:hCD8^+^ T cell ratio was reduced by 54% in hPBMC + Ctrl Ab (*P* = 0.05) and by 39% in hPBMC + PTCy + TOC mice (*P* = 0.56) (Figure 7e). This reduction was similar between the latter two groups (*P* = 0.93). This indicates that the presence of THP‐1 cells alters the proportion of hCD4^+^ and hCD8^+^ T cells, which is not impacted by treatment with PTCy + TOC.

In the liver, proportions of hTregs were similar between all groups (2.3–2.7%) (*P* = 0.86) (Figure 7f), as were the proportions of hCD39^+^ hTregs (65.8–76.2%) (*P* = 0.11) (Figure 7g). Proportions of hTh17 cells were also similar between all groups (16.2–18.0%) (*P* = 0.91) (Figure 7h), as were the hTh17:hTreg ratios (9.5–12.1) (*P* = 0.74) (Figure 7i). hPBMC only and hPBMC + Ctrl Ab mice had similar proportions of hTc17 cells (1.5 ± 0.4% vs 1.2 ± 0.4%) (*P* = 0.73) (Figure 7j). Comparatively, proportions of hTc17 cells in hPBMC + PTCy + TOC mice were reduced by approximately 60%; however, this was not significant (*P* = 0.61) (Figure 7j). With the exception of two outliers in the hPBMC only group, proportions of hNKT cells were low and similar between groups (2.8–16.5%) (*P* = 0.35) (Figure 7k) as were the proportions of hNK cells (0.4–1.4%) (*P* = 0.35) (Figure 7l). Together, these results indicate that treatment with PTCy + TOC does not impact the proportions of these leukocyte subsets in this model.

## Discussion

Donor HSCT treats leukaemia by inducing GVL immunity and is an effective cellular immunotherapy for hematologic malignancies. However, a common side effect is the development of GVHD, which results in severe damage to non‐target tissues and is fatal in 15–30% of cases.^3^ The current study developed a humanised mouse model of GVL immunity to examine the impact of using different donors and GVHD treatments on GVL responses. This study found that the GVL response is donor‐specific, and donors with reduced liver hCD4^+^:hCD8^+^ T cell ratios and increased hTh17:hTreg ratios could mediate effective GVL responses, whereas the donor with lower hCD8^+^ and hTh17 proportions showed reduced capacity to mediate GVL. Further, there was increased hIL‐10 and hIFNγ in donors mediating GVL responses. We next examined whether GVHD therapies including PTCy alone or combination therapy using PTCy and TOC impacts these GVL responses. Here, PTCy did not compromise GVL immunity, showing similar changes to immune subsets and no impact on circulating cytokines. Further, PTCy combined with TOC, which can further delay GVHD onset,^23^ did not impact GVL responses, showed reduced histopathology in the liver and lung and had minimal impact on T cell subsets.

A GVL response to THP‐1 cells was observed in mice injected with hPBMCs from two donors. These mice had higher hCD3^+^ T cell proportions and lower hCD4:hCD8^+^ T cell ratios than those that received hPBMCs from the donor that did not elicit a GVL response. This implies that T cells, and hCD8^+^ T cells in particular, may have a central role in generating the GVL response in this model. Other groups have investigated GVL responses against THP‐1 cells in humanised mice; however, hCD4^+^ and hCD8^+^ T cell proportions were not reported.^40^, ^41^, ^42^ In the clinical setting, both CD4^+^ and CD8^+^ T cells contribute to GVL effects.^43^ Removal of either CD4^+^ or CD8^+^ T cells from a graft has shown to compromise GVL reactivity, indicating that the presence of both subsets is required for optimal GVL responses.^44^

Notably, mice injected with hPBMCs that did not elicit a GVL response had increased proportions of splenic hTregs. It has previously been shown that a higher number of Tregs is associated with tumor relapse and tumor growth in both mouse and human studies.^45^, ^46^ However, other studies suggest that Tregs have no impact on the GVL response when utilised as a treatment for GVHD.^47^, ^48^ Mice injected with hPBMCs that did elicit a GVL response had greater proportions of hTh17 cells and a higher hTh17:hTreg ratio compared to mice injected with hPBMCs that did not elicit a GVL response. This may suggest that Th17 cells are involved in GVL responses in this model. Conversely, a study by Delens *et al*. contradicts these findings, suggesting that Th17 cells do not contribute to GVL responses against THP‐1 cells. However, this study differs from the current study as mice were irradiated, co‐injected with PBMCs and Th17‐polarised cells and injected subcutaneously with THP‐1 cells.^40^ Furthermore, differences between the aforementioned study and the current study could be because of differences between donors or the transformation of THP‐1 cells, the latter or which was not assessed in either study. Nevertheless, the differences observed in the current study in the hTh17:hTreg ratio in relation to the GVL response may relate to the increase in hTregs rather than the reduction in hTh17 cells.

In the current study, the concentration of hIL‐10 and hIFNγ was elevated in the sera of mice who received hPBMCs from the donors that did elicit a GVL response. In the context of acute myeloid leukaemia, patients with higher plasma IL‐10 concentrations have prolonged overall survival and greater event‐free survival and remission rates.^49^, ^50^ Furthermore, *in vitro* experiments found that IL‐10 inhibited acute myeloid leukaemia blast proliferation and the production of pro‐leukaemic cytokines.^51^ IFNγ is a pleiotropic cytokine produced by multiple cell types including activated T cells, NKT cells and NK cells. Moreover, IFNγ has been shown to facilitate GVL responses in IL‐12‐treated donor HSCT recipients^52^ and has been shown to facilitate CD8^+^ T cell‐mediated GVL responses.^53^

Low and similar proportions of hCD33^+^ THP‐1 leukaemia cells were observed in both hPBMC and hPBMC + PTCy mice, indicating that PTCy does not impact GVL responses in this model. These results are similar to a recent study which found that the GVL response was maintained in NSG‐HLA‐A2/HHD mice injected with 2 × 10^7^ hPBMCs, THP‐1 cells and a single 100 mg kg^−1^ injection of cyclophosphamide.^16^ Conversely, high rates of leukaemia relapse were observed when a 50 mg kg^−1^ dose of cyclophosphamide was given on days 3 and 4 in initial clinical studies.^14^, ^15^ Despite its use clinically, the mechanism by which PTCy impacts alloreactive donor T cells and reduces GVHD is poorly understood, with some studies suggesting depletion of effector T cells,^54^ effector T cell dysfunction^11^ or enhancement of Tregs^55^ as its mechanism of action. As such, further exploration into the precise mechanism by which PTCy reduces GVHD and its role in GVL responses is required.

Notably, in the current study, PTCy did not impact the engraftment of hCD45^+^ leukocytes or hCD3^+^ T cells. However, PTCy reduced hCD4^+^:hCD8^+^ T cell ratios. A high CD4^+^:CD8^+^ T cell ratio is a clinical indicator of increased risk of GVHD and mortality following donor HSCT.^56^ We and others have previously shown that an increased hCD4^+^:hCD8^+^ T cell ratio correlates with worsened GVHD severity in NSG mice.^31^, ^40^ However, results from our studies in humanised mouse models of GVHD are conflicting, which may be because of different time points being examined and different donors used. In one study, the hCD4^+^:hCD8^+^ ratio was increased at endpoint in mice injected with 2 × 10^7^ hPBMCs and treated with PTCy compared to mice treated with PBS.^12^ In a second study, the hCD4^+^:hCD8^+^ ratio was not impacted in mice injected with 2 × 10^7^ hPBMCs and treated with PTCy, using the same dose and route of administration, either early in disease or at endpoint.^23^ Future studies should seek to inject isolated hCD4^+^ or hCD8^+^ T cells into mice to assess the impact of each T cell subset in mediating GVL responses and to assess the impact of PTCy on each of these subsets alone.

To our knowledge, the impact of PTCy combined with TOC on GVL immunity has not been previously assessed either clinically or in mouse models. In the current study, GVL immunity was maintained and there was no impact on the proportions of immune cell subsets. We have previously shown that TOC delays the onset of GVHD in humanised mice treated with PTCy while increasing the proportion of Tregs and reducing the proportion of NK cells.^23^ While the delay in GVHD onset was again observed, and reduced liver and lung GVHD was found in the current study, there were no differences observed in Tregs or NK cells, nor any other immune cell subsets. Importantly, this provides evidence that this combination therapy PTCy and TOC can be used to treat GVHD without compromising GVL immunity or other immune responses.

The current study has some limitations. The grading system used to score the mice does not allow us to distinguish specifically between GVHD, GVL responses or leukaemia, thus clinical disease may be due to one or more of these events. Further, the current study assessed cell proportions but not absolute numbers of immune cell subsets. Thus, the ability of PTCy to impair proliferation or induce death of T cells or subsets cannot be deduced entirely. Finally, to avoid the impact of PTCy, THP‐1 cells were given after PTCy treatment. Therefore, this does not fully replicate donor HSCT, where there is likely to be residual malignant cells present following pre‐conditioning radiation and/or chemotherapy and prior to transplantation.

To conclude, this study determined that GVL responses to THP‐1 cells are donor‐specific in this humanised mouse model. Notably, this study demonstrated that treatments that reduce GVHD including PTCy alone or combined with TOC have no impact on GVL responses. While the current study assessed GVL responses at endpoint by assessing leukaemia cells present in tissues, future studies which employ the use of bioluminescent imaging to track tumor burden over time may provide further insight into the mechanisms of GVL responses.

## Methods

### Mice

Animal procedures were conducted as approved by the University of Wollongong Animal Ethics Committee (Wollongong, Australia) (AE20/05). Female NOD‐*scid*‐IL2Rγ^null^ (NSG) mice aged 5–7 weeks were obtained from the Animal Resource Centre (Canning Vale, Australia) or Australian BioResources (Moss Vale, Australia) and were acclimatised for at least 2 weeks before commencing experimental work. Mice were housed in individually ventilated cages with high‐efficiency particulate absorbing filters (Tecniplast, Lane Cove, Australia) under a 12‐h light/12‐h dark cycle. Mice were housed four per cage, given autoclaved water and irradiated food *ad libitum* and were provided with enrichments.

### THP‐1 leukaemia cells

Human monocytic THP‐1 cells derived from an acute monocytic leukaemia patient (American Type Culture Collection; Rockville, USA) were maintained in RPMI‐1640 medium (Life Technologies, Carlsbad, USA) containing 10% foetal calf serum (FCS) (Thermo Fisher Scientific, Waltham, USA) and 2 mM GlutaMAX (Thermo Fisher Scientific) at 37°C with 5% CO_2_. Cells were passaged twice weekly and routinely tested for mycoplasma by the Illawarra Health and Medical Research Institute technical officers using the MycoAlert Test Kit (Lonza, Basel, Switzerland). Short tandem repeat analysis (Garvan Molecular Genetics, Sydney) revealed that these cells were 100% identical to reference THP‐1 cells. On the day of injection into NSG mice, THP‐1 cells were washed twice with Dulbecco's phosphate‐buffered saline (PBS) (Thermo Fisher Scientific) (300 × *g* for 5 min) and resuspended at 1 × 10^7^ cells mL^−1^.

### Graft‐versus‐leukaemia humanised mouse model

Experiments involving human blood were approved by the University of Wollongong Human Ethics Committee (HE 12/290). Peripheral blood was collected from healthy, consenting donors (*n* = 4, 50% male/female and 23–31 years of age) and human peripheral blood mononuclear cells (hPBMCs) were isolated by density gradient centrifugation as described.^57^ Isolated hPBMCs were washed with sterile PBS (Thermo Fisher Scientific) (440 × *g*, 5 min) and resuspended at 10 × 10^7^ cells mL^−1^ in PBS for injection into NSG mice. To establish the GVL model, on day 0, NSG mice were injected intraperitoneally (i.p.) with 1 × 10^7^ hPBMCs or PBS. On day 14 post‐hPBMC injection, mice were injected intravenously (i.v.) with 1 × 10^6^ THP‐1 cells, which was determined to achieve sufficient leukaemia engraftment while minimising side effects (Supplementary figure 1). In subsequent studies, humanised NSG mice were also injected i.p. with 33 mg kg^−1^ cyclophosphamide (Sigma‐Aldrich, St Louis, USA) or PBS alone on days 3 and 4 post‐hPBMC injection, or combined with tocilizumab (TOC: Chugai Pharmaceutical, Tokyo, Japan), a recombinant humanised anti‐human IL‐6 receptor monoclonal antibody of the immunoglobulin G1k subclass,^58^ or a control antibody (rat IgG) (Sigma‐Aldrich), administered twice weekly for 28 days as previously described.^23^ All mice were monitored for clinical signs of GVHD and/or leukaemia for up to 42 days using a standardised grading system (Table 1). At endpoint, mice were euthanised by slow‐fill CO_2_ before tissues were collected. Mice that did not engraft (< 5% hCD45^+^ leukocytes of total leukocytes; *n* = 1 in the PTCy study and *n* = 2 in the PTCy + TOC study) were excluded from all analyses.

### Immunophenotyping

Cells were isolated from spleens and livers as described^24^ prior to live/dead staining with Zombie Near Infrared (NIR) dye (BioLegend, San Diego, USA). Cells were subsequently incubated with fluorochrome‐conjugated monoclonal antibodies (Supplementary table 1) in PBS containing 2% FCS for 10 min, on ice protected from light. Samples were washed in PBS (300 × *g* for 5 min) and then resuspended in 300 μL PBS for analysis. Data were collected using an LSRFortessa X‐20 flow cytometer (BD Biosciences) using the appropriate band‐pass filters (Supplementary table 1) and analysed as previously described.^23^

### LEGENDplex

Serum was isolated from mouse blood collected via cardiac puncture as described^59^ and stored at −80°C. Concentrations of human interleukin (IL)‐2, IL‐6, IL‐10, interferon‐gamma (IFNγ) and tumor necrosis factor‐alpha (TNFα) in mouse sera were determined using a 5‐plex Th1 panel LEGENDplex™ kit (BioLegend) per the manufacturer's instructions. Data were collected using an Attune™ NxT acoustic focusing cytometer and Attune NxT software v3.1 (Invitrogen, Waltham, USA) and analysed using LEGENDplex™ data analysis software v8.0. Cytokines below the detection limit were assigned half the concentration of the lowest standard and cytokines above the detection limit were set to the limit for statistical analyses.

### Haematoxylin and eosin staining

Samples of liver and lung collected from euthanised mice were fixed overnight in neutral buffered formalin (10%) (Sigma‐Aldrich). Tissues were processed using an ASP200S tissue processor (Leica, Buffalo Grove, USA) and embedded in paraffin wax. Tissues were sectioned (3 μm) using an RM2255 microtome (Leica) and stained with haematoxylin and eosin (POCD, Artarmon, Australia). Histological damage was assessed using a DM750 inverted light microscope (Leica) with the 20× objective and images were captured using Leica Application Suite Software v4.7. Liver damage was graded in a blinded fashion using a standardised grading system (using grades 0–4) as described.^60^ Lung damage was assessed by blinded area measurements of open alveoli space using Fiji^61^ and quantified as a percentage of the total lung area measured.^62^

### Data presentation and statistical analysis

Data are presented as the mean ± standard error of the mean (SEM). A Shapiro–Wilk test was applied to test for normality prior to a two‐tailed Student's *t*‐test (parametric) or Mann–Whitney *U*‐test (non‐parametric) to determine statistical differences between single comparisons. A one‐way analysis of variance (ANOVA) (parametric) or Kruskal‐Wallis (non‐parametric) test was applied with Tukey's *post hoc* test for multiple comparisons. A two‐way ANOVA was applied for comparisons of mouse weight and clinical score, with weights and scores from mice euthanised before day 42 carried forward, and a log‐rank (Mantel‐Cox) test was applied for mouse survival comparisons. Statistical analysis and graphs were generated using GraphPad Prism software v9.1.1 (GraphPad Software; La Jolla, USA). Differences where *P* < 0.05 were considered statistically significant for all analyses.

## Author contributions

**Chloe Sligar:** Data curation; formal analysis; investigation; visualization; writing – original draft; writing – review and editing. **Ellie Reilly:** Investigation; visualization; writing – review and editing. **Peter Cuthbertson:** Investigation; writing – review and editing. **Kara L Vine:** Investigation; writing – review and editing. **Katrina M Bird:** Investigation. **Amal Elhage:** Investigation; writing – review and editing. **Stephen I Alexander:** Resources. **Ronald Sluyter:** Conceptualization; funding acquisition; methodology; project administration; resources; supervision; writing – review and editing. **Debbie Watson:** Conceptualization; funding acquisition; methodology; project administration; resources; supervision; writing – review and editing.

## Conflict of interest

The authors declare no conflict of interest.

## Funding information

This research was funded by Cancer Council NSW (grant number: RG 19‐12) (DW), the University of Wollongong RevITAlise (RITA) Research Grant Scheme (grant number: T021) (DW and RS) and the Australian Government Research Training Program Scholarships (CS, PC and AE).

## Supporting information


Supplementary figure 1
Supplementary figure 2
Supplementary figure 3
Supplementary figure 4
Supplementary figure 5
Supplementary table 1

## Data Availability

The data that support the findings of this study are available from the corresponding author upon reasonable request.
