# Supplementary material for: Graft‐versus‐leukaemia immunity is retained following treatment with post‐transplant cyclophosphamide alone or combined with tocilizumab in humanised mice
Source: Clin Transl Immunology. 2024 Mar 15;13(3):e1497. doi: 10.1002/cti2.1497 (PMC10941522; doi:10.1002/cti2.1497)
Supplement: Supplementary file 1 — Supplementary figure 1 Supplementary figure 2 Supplementary figure 3 Supplementary figure 4 Supplementary figure 5 Supplementary table 1 [file CTI2-13-e1497-s001.pdf]

Graft-versus-leukaemia immunity is retained following treatment with post-transplant cyclophosphamide alone or combined with tocilizumab in humanised mice

Chloe Sligar[1,2], Ellie Reilly[1,2], Peter Cuthbertson[1,2], Kara L.Vine[1,2], Katrina M. Bird[1,2], Amal Elhage[1,2], Stephen I. Alexander[3], Ronald Sluyter[1,2*], Debbie Watson[1,2*]

*Equal authors

[1]Molecular Horizons and School of Chemistry and Molecular Bioscience, University of Wollongong, Wollongong, NSW 2522, Australia; [2]Illawarra Health and Medical Research Institute, Wollongong, NSW 2522, Australia, [3]The Children's Hospital at Westmead, Westmead, NSW 2145, Australia.

Running title: GVL immunity retained with PTCy and Tocilizumab

Supplementary File

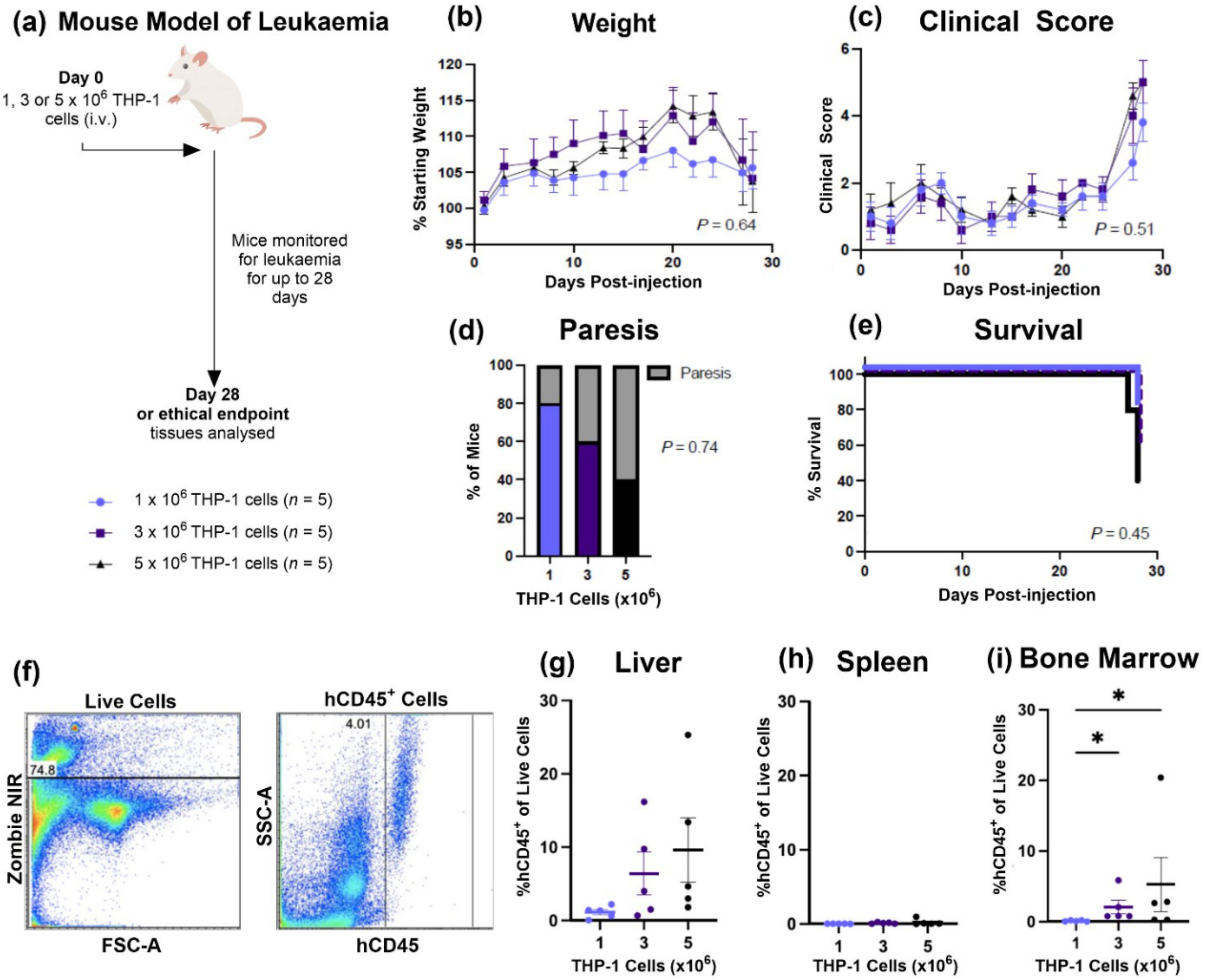

Supplementary figure 1: Weight loss, clinical score and survival are similar between NSG mice injected with different doses of THP-1 cells and THP-1 cells primarily engraft in the liver. (a) Schematic overview of leukaemia mouse model. NOD-*scid*-IL2Rγ^{null} (NSG) mice were injected intravenously (i.v.) with 1 x 10^6, 3 x 10^6 or 5 x 10^6 THP-1 cells on day 0. Mice were monitored at least thrice weekly for clinical signs of leukaemia including **(b)** weight, **(c)** clinical score, **(d)** paresis and **(e)** survival over 28 days. At endpoint, livers, spleens and bone marrow were assessed by flow cytometry using **(f)** a consistent gating strategy. Live cells were gated based on Zombie NIR staining and THP-1 cells were identified using side scatter area (SSC-A) and hCD45. **(g-i)** Proportions of hCD45$^+$ THP-1 cells in the **(g)** liver, **(h)** spleen and **(i)** bone marrow are shown as a percentage of total live cells. Data are presented as the mean ± SEM. Symbols represent individual mice. Significance was analysed using **(b, c)** two-way ANOVA, **(d)** Chi-squared, **(e)** Mantel Cox and **(g, h, i)** Kruskal-Wallis tests. $P < 0.05$ (*).

Supplementary table 1: Fluorochrome-conjugated monoclonal antibodies used for immunophenotyping human cell subsets

Antibodies*	Clone	Conjugated fluorophore	Emission band-pass filter
Mouse anti-human CD45	HI30	Fluorescein isothicyanate	525/15
Mouse anti-human CD33	P67.6	R-phycoerythrin (PE)	780/60
Mouse anti-human CD127	HIL-7R-M21	Brilliant violet (BV) 421	450/50
Mouse anti-human CD161	DX12	BV605	610/20
Mouse anti-human CD3	UCHT1	BV711	710/50
Mouse anti-human CD25	M-A251	PE	586/15
Mouse anti-human CD39	TU66	Allophycocyanin	670/30
Mouse anti-human CD4	SK3	Perdinin-chlorophyll-protein (PerCP) cyanine 5.5	695/40
Mouse anti-human CD8	RPA-T8	PE-cyanine 7	780/60
Rat anti-mouse CD45	30-F11	PerCP	695/40

*Obtained from BD Biosciences (San Jose, USA).

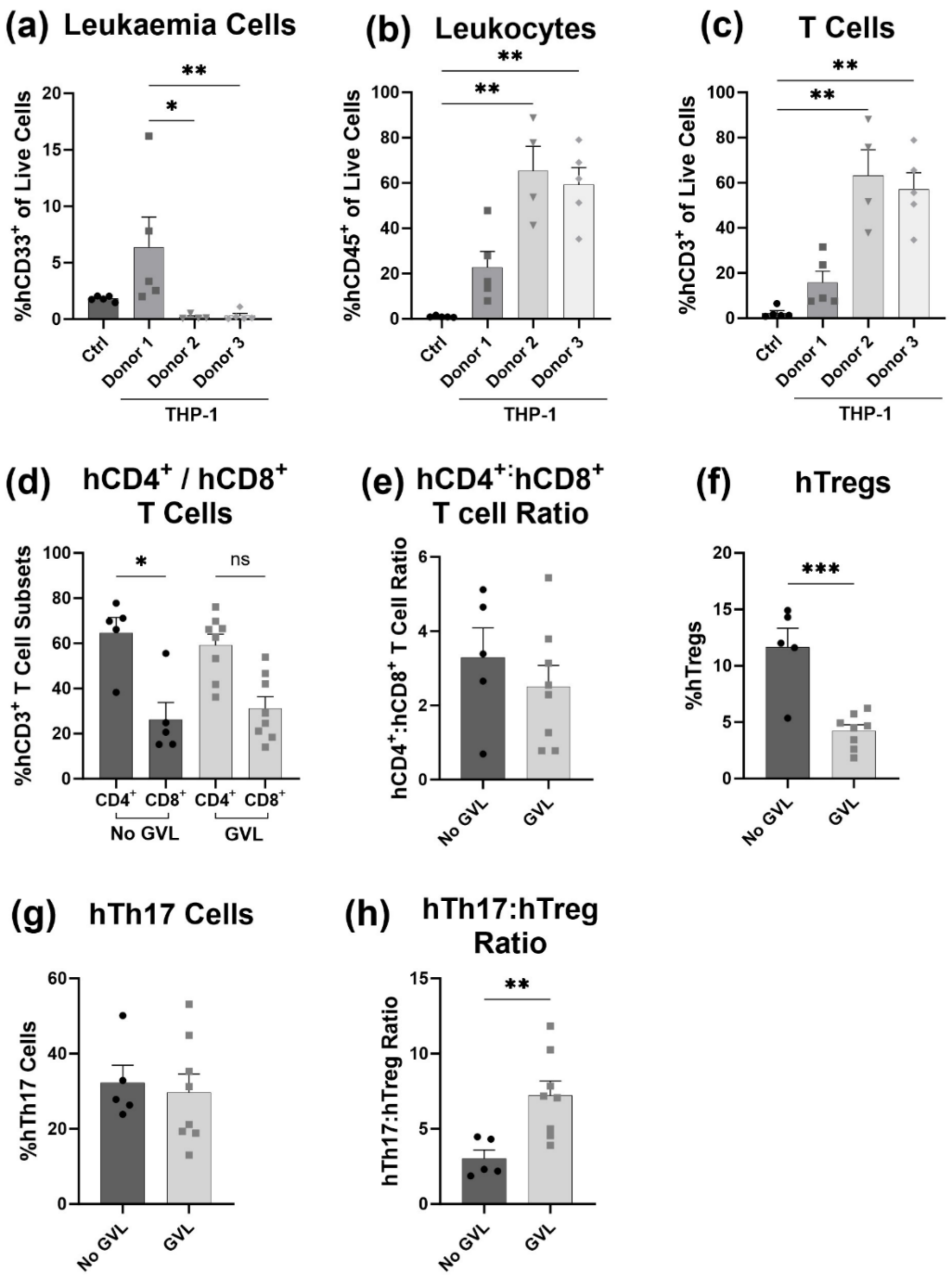

Supplementary figure 2: NSG mice injected with hPBMCs from donor unable to elicit a GVL response have a greater proportion of splenic hTregs and a lower splenic hTh17:hTreg ratio. Spleens from NOD-*scid*-IL2Rγ^{null} (NSG) mice injected intraperitoneally (i.p.) with 1 x 10^7 human peripheral blood mononuclear cells (hPBMCs) from donors 1 (D1), 2 (D2) and 3 (D3) or PBS on day 0 and intravenously (i.v.) with 1 x 10^6 THP-1 cells on day 14 were collected at day 42 or ethical endpoint and analysed by flow cytometry. Live cells were gated based on Zombie NIR staining (not shown) before **(a)** $hCD33^+$ leukaemia, **(b)** $hCD45^+$ leukocytes and **(c)** $hCD3^+$ T cells were gated using the gating strategy (as shown in Figure 2**a).** An established gating strategy[22] was used to identify and analyse **(d)** $hCD4^+$ and $hCD8^+$ T cells, **(e)** $hCD4^+$:$hCD8^+$ T cell ratio, **(f)** $hCD4^+hCD25^+hCD127^{low}$ regulatory T cells (hTregs), **(g)** $hCD4^+hCD161^+hCD39^+$ T helper 17 (hTh17) cells and **(h)** hTh17:hTreg ratio. Data from mice injected with hPBMCs from the donor (D1) that was unable to elicit a graft-versus-leukaemia (GVL) response (no GVL) was compared to those injected with hPBMCs from donors (D2 and D3) that did establish a GVL response (GVL). Data are presented as the mean ± SEM. Symbols represent individual mice. Significance was determined using **(a, b, c)** Kruskal-Wallis or **(d, e, f, g, h)** unpaired Student's *t*-tests. $P <$ 0.01 (**), $P < 0.05$ (*), not significant (ns).

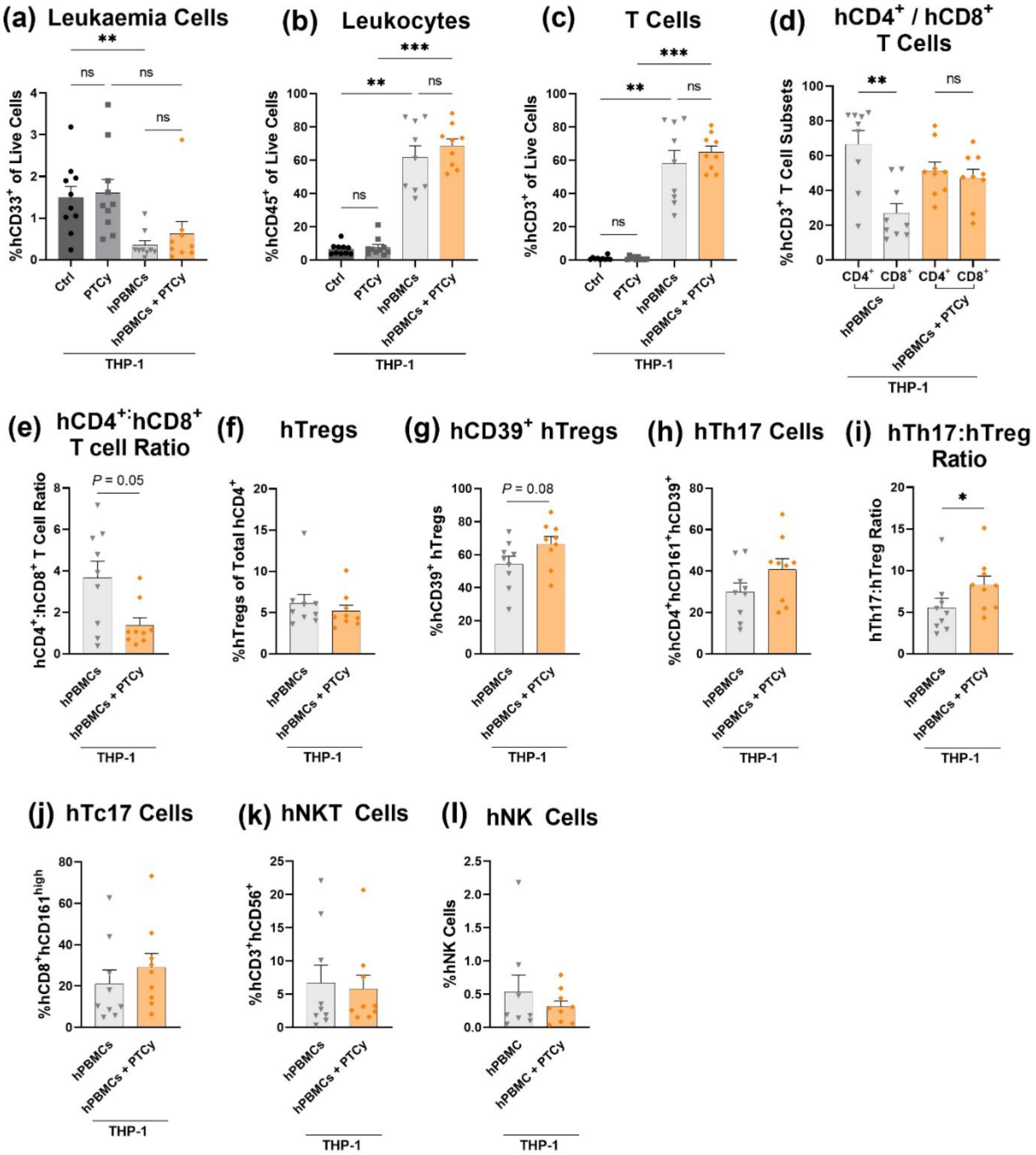

Supplementary figure 3: PTCy reduces the hCD4^{+}:hCD8^{+} T cell ratio and increases the hTh17:hTreg ratio in the spleens of NSG mice injected with hPBMCs and THP-1 cells. Spleens from NOD-*scid*-IL2Rγ^{null} (NSG) mice injected intraperitoneally (i.p.) with 2 x 10^{7} human peripheral blood mononuclear cells (hPBMCs) (n = 2 donors; D2 and D3) or PBS on day 0, i.p. with cyclophosphamide (33 mg.kg^{-1}) (PTCy) or PBS on days 3 and 4, intravenously (i.v.) with 1 x 10^{6} THP-1 cells on day 14 were examined by flow cytometry. Live cells were gated based on Zombie NIR staining (not shown), leukaemia cells and T cells were gated using hCD33 and hCD3, respectively, and leukocytes were gated using side-scatter area (SSC-A) and hCD45. Proportions of **(a)** hCD33^{+} leukaemia cells, **(b)** hCD45^{+} leukocytes and **(c)** hCD3^{+} T cells were gated (as shown in Figure 2**a**) and analysed as a proportion of live cells. An established gating strategy[22] was used to identify **(d)** hCD4^{+} and hCD8^{+} T cell subsets, **(e)** hCD4^{+}:hCD8^{+} T cell ratio, (f) hCD4^{+}hCD25^{+}hCD127^{low} regulatory T cells (hTregs), **(g)** hCD39^{+} hTregs, **(h)** hCD4^{+}hCD161^{+}hCD39^{+} T helper 17 (hTh17) cells, **(i)** hTh17:hTreg ratio, **(j)** hCD8^{+}hCD161^{high} (hTc17) cells, **(k)** hCD3^{+}hCD56^{+} natural killer T (hNKT) cells and **(l)** hCD3^{-}hCD56^{+} natural killer (hNK) cells. Data are presented as the mean ± SEM. Symbols represent individual mice. Data are from two independent experiments. Significance was determined using either **(a, b, c, d)** Kruskal-Wallis, **(g, h)** unpaired Student's *t*- or **(e, f, i, j, k, l)** Mann-Whitney *U*-tests. $P < 0.001$ (***), $P < 0.01$ (**), $P < 0.05$ (*).

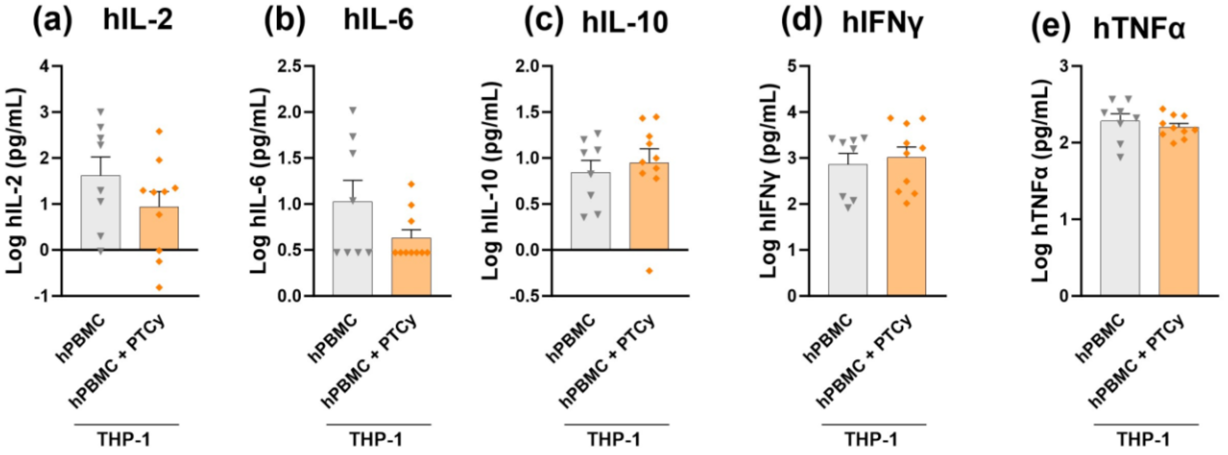

Supplementary figure 4: PTCy does not impact the concentration of human T cell cytokines in the sera of NSG mice injected with hPBMCs and THP-1 cells. At day 42 or ethical endpoint, sera were isolated from the blood of NOD-*scid*-IL2Rγ^{null} (NSG) mice injected intraperitoneally (i.p.) with 2 x 10^7 human peripheral blood mononuclear cells (hPBMCs) (n = 2 donors; D2 and D3) or PBS on day 0, i.p. with cyclophosphamide (33 mg.kg^{-1}) (PTCy) or PBS on days 3 and 4, intravenously (i.v.) with 1 x 10^6 THP-1 cells on day 14. The concentration of human (h) **(a)** IL-2, **(b)** IL-6, **(c)** IL-10, **(d)** IFNγ and **(e)** TNFα was assessed using a LEGENDplex assay. Data are presented as the mean ± SEM. Symbols represent individual mice. Data are from two independent experiments. Significance was tested using either **(a, e)** unpaired Student's *t*- or **(b, c, d)** Mann-Whitney *U*-tests.

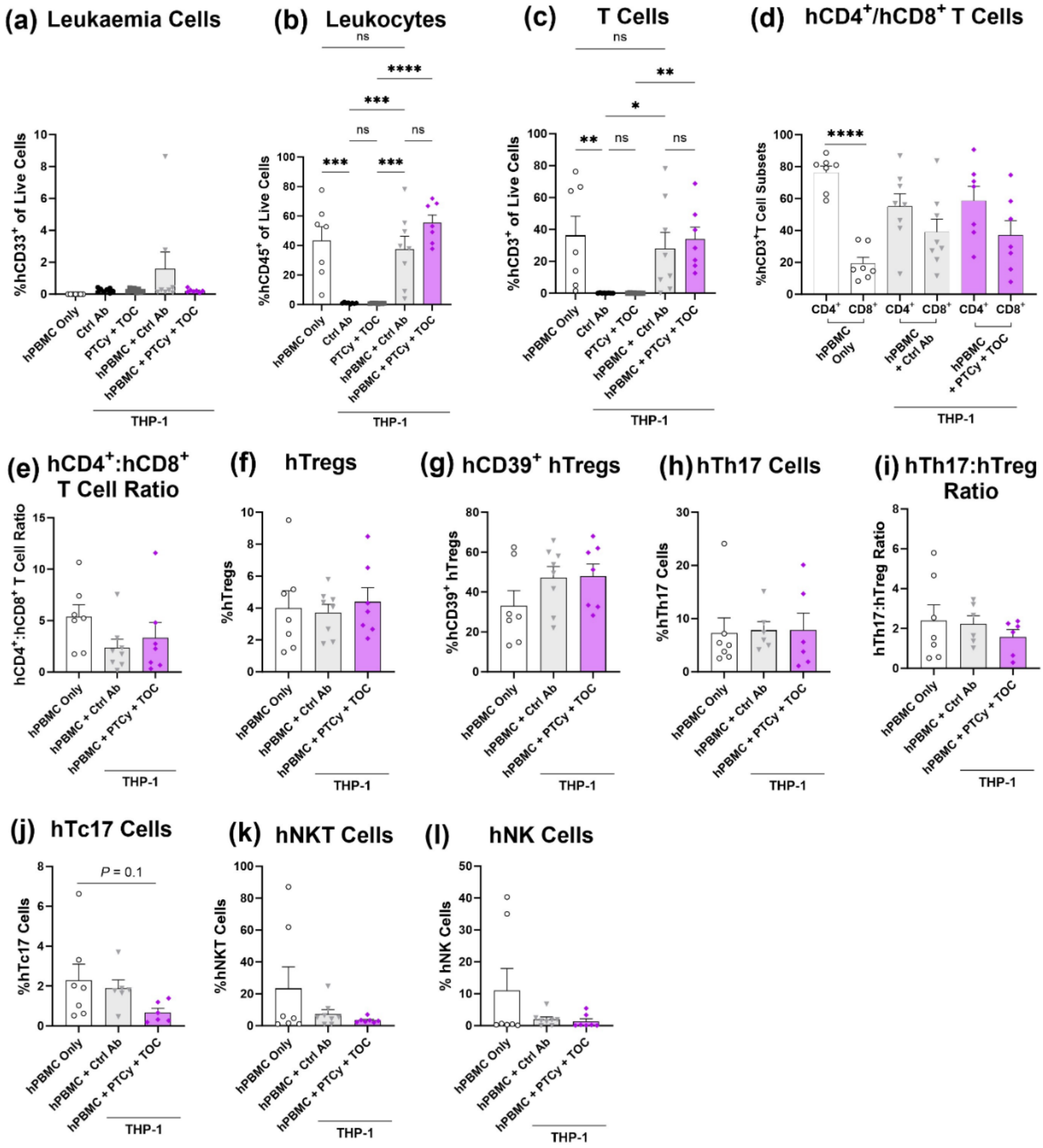

Supplementary figure 5: PTCy + TOC does not influence immune cell subsets in the spleens of NSG mice injected with hPBMCs and THP-1 cells. Spleens from NOD-*scid*-IL2Rγ^{null} (NSG) mice injected intraperitoneally (i.p.) with 2 x 10^7 human peripheral blood mononuclear cells (hPBMCs) (n = 2 donors; D2 and D3) or PBS on day 0, i.p. with tocilizumab (TOC) or control antibody (Ctrl Ab) (25 mg.kg^{-1}) twice weekly for 28 days, i.p. with cyclophosphamide (33 mg.kg^{-1}) (PTCy) or PBS on days 3 and 4 and i.v. with 1 x 10^6 THP-1 cells on day 14 were examined by flow cytometry. Live cells were gated based on Zombie NIR staining (not shown), leukaemia cells and T cells were gated using hCD33 and hCD3, respectively, and leukocytes were gated using side-scatter area (SSC-A) and hCD45. Proportions of **(a)** hCD33$^+$ leukaemia cells, **(b)** hCD45$^+$ leukocytes and **(c)** hCD3$^+$ T cells were gated and analysed as a proportion of live cells. An established gating strategy[22] was used to identify **(d)** hCD4$^+$ and hCD8$^+$ T cells, **(e)** hCD4$^+$:hCD8$^+$ T cell ratio, **(f)** hCD4$^+$hCD25$^+$hCD127^{low} regulatory T cells (hTregs), **(g)** hCD39$^+$ hTregs, **(h)** hCD4$^+$hCXCR3$^-$hCD161$^+$ T helper 17 (hTh17) cells, **(i)** hTh17:hTreg ratio, **(j)** hCD8$^+$hCD161^{high} (hTc17) cells, **(k)** hCD3$^+$hCD56$^+$ natural killer T (hNKT) cells and **(l)** hCD3$^-$hCD56$^+$ natural killer (hNK) cells. Data are presented as the mean ± SEM. Symbols represent individual mice. Data are from two independent experiments. Significance was determined using either **(b, d, f, g, i, j)** one-way ANOVA or **(a, c, e, h, k, l)** Kruskal-Wallis tests. $P < 0.0001$ (****), $P < 0.001$ (***), $P < 0.01$ (**), $P < 0.05$ (*).
